# JR5558 mice are a reliable model to investigate subretinal fibrosis

**DOI:** 10.1038/s41598-024-66068-z

**Published:** 2024-08-13

**Authors:** Yashar Seyed-Razavi, So-Ra Lee, Jiawen Fan, Weiyong Shen, Elisa E. Cornish, Mark C. Gillies

**Affiliations:** 1 Save Sight Institute, Discipline of Ophthalmology, Sydney Medical School, The University of Sydney, Sydney, NSW 2000 Australia; 2 Centre for Vision Research, Westmead Institute for Medical Research, Faculty of Medicine and Health, Sydney University, Sydney, Westmead, NSW 2145 Australia

**Keywords:** Retina, Fibrosis, Lesions, Extracellular matrix, Gliosis, Müller cells, Retina, Experimental models of disease

## Abstract

Subretinal fibrosis is a major untreatable cause of poor outcomes in neovascular age-related macular degeneration. Mouse models of subretinal fibrosis all possess a degree of invasiveness and tissue damage not typical of fibrosis progression. This project characterises JR5558 mice as a model to study subretinal fibrosis. Fundus and optical coherence tomography (OCT) imaging was used to non-invasively track lesions. Lesion number and area were quantified with ImageJ. Retinal sections, wholemounts and Western blots were used to characterise alterations. Subretinal lesions expand between 4 and 8 weeks and become established in size and location around 12 weeks. Subretinal lesions were confirmed to be fibrotic, including various cell populations involved in fibrosis development. Müller cell processes extended from superficial retina into subretinal lesions at 8 weeks. Western blotting revealed increases in fibronectin (4 wk and 8 wk, *p* < 0.001), CTGF (20 wks, *p* < 0.001), MMP2 (12 wks and 20 wks *p* < 0.05), αSMA (12 wks and 20 wks *p* < 0.05) and GFAP (8 wk and 12 wk, *p* ≤ 0.01), consistent with our immunofluorescence results. Intravitreal injection of Aflibercept reduced subretinal lesion growth. Our study provides evidence JR5558 mice have subretinal fibrotic lesions that grow between 4 and 8 weeks and confirms this line to be a good model to study subretinal fibrosis development and assess treatment options.

## Introduction

There is an urgent unmet need to identify ways to prevent subretinal fibrosis in neovascular age-related macular degeneration (nAMD). Age-related macular degeneration (AMD) is a progressive disease estimated to affect 288 million people by 2040^1^. The hallmarks of “wet” AMD include the growth and penetrance of abnormal new vessels from the choriocapillaris (choroidal neovascularization (CNV)), resulting in vascular leakage and haemorrhage beneath the RPE or photoreceptors, eventually leading to retinal atrophy and subretinal scar formation^2^. The gold-standard in treatment for nAMD is intravitreal (IVT) injection of vascular endothelial growth factor (VEGF) inhibitors to suppress CNV by decreasing vascular leakage and haemorrhage to improve vision acuity. While VEGF inhibitors are effective antiangiogenic agents, they are unable to prevent subretinal fibrotic scar formation in many eyes with nAMD^3^.

Fibrosis is pathological wound healing in response to chronic injury within the tissue that results in a robust inflammatory process and excessive abnormally ordered extracellular matrix (ECM) deposition^4^. The location of ECM depositions during the healing process affects correct repair and restoration of functionality^5,6^. In the eye, fibrovascular scarring may be formed due to inflammation, neovascularisation or neurodegeneration, can be observed in proliferative vitreoretinopathy (PVR), proliferative diabetic retinopathy (PDR), neovascular (“wet’) age-related macular degeneration (nAMD) and inherited retinal degenerations (IRDs)^7^. Excessive deposition of collagenous ECM proteins within subretinal lesions in retinal fibrosis involves various cell populations, including Müller cells, microglia, fibroblasts, myofibroblast-like cells, and the retinal pigment epithelium (RPE)^8–11^. The disruption of retinal architecture and normal cell–cell relationships in retinal fibrosis in turn leads to damage to the RPE, photoreceptors, Bruch’s membrane, and choroidal vessels. Clinically, this disruption appears as a well demarcated white-yellow lesion with a solid opaque appearance in fundus images, as well as a thick uniformly hyperreflective area with distinct borders below the neural retina in optical coherence tomography (OCT) scans^12^. OCT angiography consistently detects blood flow within subretinal fibrotic scars, highlighting the fibrovascular nature of these lesions^13^. Currently there is no approved preventative or treatment for subretinal fibrosis, which is irreversible once established.

Cells undergoing epithelial to mesenchymal transition (EMT), a process where epithelial cells lose the expression of E-Cadherin^14^, develop a more mesenchymal phenotype than their parent epithelial cells by expressing certain proteins, including N-cadherin, vimentin and fibronectin^15,16^, and alter their morphology—which also allows better tissue migration^17^. EMT is accepted as a driving force behind tissue fibrosis^18^. Although the underlying mechanisms of ECM deposition and origin of myofibroblasts in retinal fibrotic scars remains unclear, studies have suggested that transdifferentiation of RPE cells to myofibroblasts^11^ and gliosis from Müller cells, microglia and astroglia^19,20^ play a role in the development of subretinal fibrosis.

Whilst animal models may not present all the features of nAMD in humans, they allow for intravital analysis of the early development and progression of subretinal fibrosis. Animal models of retinal fibrosis include laser-induced subretinal fibrovascular lesions^21^, Müller cell disruption induced neovascularization leading to subretinal fibrosis^20^ and subretinally injected macrophage-rich peritoneal exudate cells^22^. A common factor in all these models is that they possess a degree of invasiveness that is not typical of fibrosis progression in nAMD, which involves CNV. The laser-induced choroidal neovascularisation model, the most prevalent model to assess angiogenic activity and vascular permeability^23^, involves acute retinal burn injury to the photoreceptors, RPE, and choriocapillaris to induce angiogenesis and retinal scar tissue. JR5558 mice develop multifocal and bilateral spontaneous CNV and neovascular lesions that result in local gliosis and focal photoreceptor dysfunction that is more similar to the human retinal condition^24,25^.

In this study, we have investigated the development of subretinal fibrovascular lesions in JR5558 mice strain by characterising differential expression of retinal and choroidal ECM, changes in the RPE, neovascularisation, photoreceptor damage and the development of fibrotic tissues in the subretinal space. Our findings indicate that JR5558 mice are a good model to investigate underlying mechanisms of subretinal fibrosis and could be useful for developing novel therapies for preventing it.

## Results

### JR5558 mice develop subretinal hyperreflective lesions

**Figure 1:**
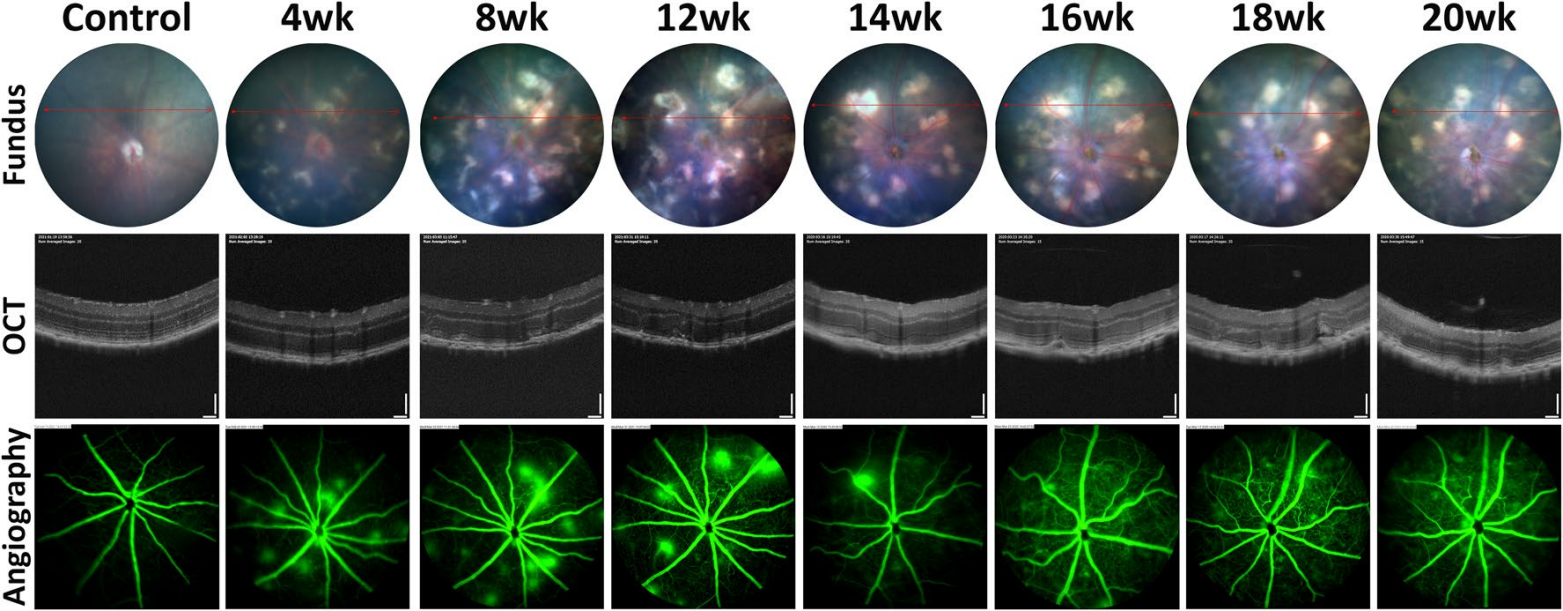
Subretinal lesions and vascular leak in the retina of JR5558 mice. Fundus photography, OCT imaging and fluorescein-angiography performed on JR5558 mice reveal subretinal yellow lesion-like areas contain hyperreflective subretinal material and correlate with vascular leak. Bright yellow lesion-like areas continued to grow between 4 and 12 weeks, and some remain static from 8 weeks onwards. 8–12 wk, 14–16 wk and 18–20 wk timeline images presented were taken from 3 different animals, and vascular leak was not noted in all lesions. Scale bar = 50 μm.

CFP revealed that JR5558 mice developed yellow lesions located beneath the neural retina from 3 to 4 weeks (Fig. 1). Similar areas were not noted in C57BL/6J controls. These yellow lesions grew with time, with the most significant growth observed between 4 and 8 weeks. Fundus colour photography in older JR5558 mice revealed bright yellow lesion-like areas that remained static from 8 weeks onwards (Fig. 1, top row, 8–12 wk, 14–16 wk and 18–20 wk; images from 3 different animals highlighting the static nature of lesions). Analysis of OCT images across the yellow lesions (red lines in fundus images) confirmed the presence of subretinal hyperreflective lesions which were similar to areas of subretinal fibrosis seen in eyes with nAMD^26^. These subretinal lesions corresponding to the bright yellow areas noted in the fundus images over time (Fig. 1, middle row). Fluorescein angiography revealed vascular leak in most but not all lesions (Fig. 1, bottom row).

We next sought to quantify the yellow lesions at the posterior pole of JR5558 from colour fundus images over time. Lesions located within the peripapillary region were traced and their frequency and area quantified. No significant difference was noted in mean lesion number between 4 and 8 weeks (15.2 ± 1.5 vs. 16.6 ± 1.1, *p* = 0.83) and 12 weeks (16.2 ± 1.4, *p* = 0.91) (Table 1). Lesion number at 20 weeks was also similar to that observed at 4 weeks of age (15.0 ± 1.9, *p* > 0.99). Analysis of the lesion area within the peripapillary region, ensuring accurate 2D presentation and quantification, revealed changes in lesion size from 0.32 ± 0.02 mm^2^ at 4 weeks, to 0.41 ± 0.02 (*p* = 0.02) at 8 weeks, 0.38 ± 0.02 (*p* = 0.21) at 12 weeks, and 0.42 ± 0.01 (*p* = 0.04) at 20 weeks of age (Table 1).

**Table 1:** Quantification of yellow lesions in colour fundus images captured of the posterior pole from of JR5558 mice.

Timepoint	4 weeks	8 weeks	12 weeks	20 weeks
Lesion number	15.2 ± 1.5	16.6 ± 1.1	16.2 ± 1.4	15.0 ± 1.9
*p*-value	–	0.830	0.914	> 0.999
Lesion Area (mm^2^)	0.32 ± 0.02	0.41 ± 0.02*	0.38 ± 0.02	0.42 ± 0.01 *
*p*-value (vs. 4 weeks)	–	0.012	0.135	0.025

*p < 0.05 compared to 4 weeks.

Whilst lesions sometimes appeared as early as 3 weeks, we noted large variation in lesion formation between 3- and 4-week timepoint in JR5558 mice. Some animals exhibited diffuse lesions with minor disruptions in the retina layers, others had clearly defined bright lesions, whilst most had no lesions at 3 weeks.

Taken together, these findings indicate that fundus and OCT examination of JR5558 mice reveal subretinal fibrovascular lesions grow reliably and predictably between 4 and 8 weeks and become well established by 12 weeks of age.

### The retinal structure of JR5558 mice is altered with outer retinal neovascularisation and gliosis

**Figure 2:**
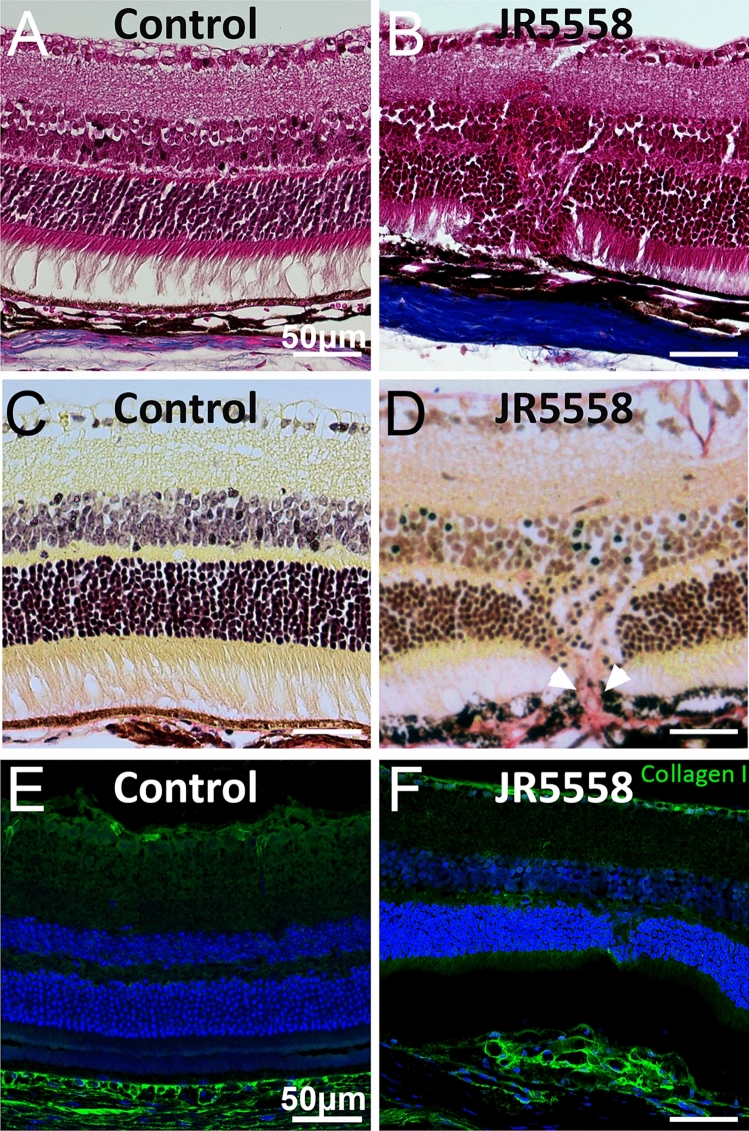
Altered morphology in the retina and subretinal space of JR5558 mice. Hematoxylin and Eosin (H&E) (**A**–**B**), Picro-Sirius Red (PSR) (**C**–**D**) and Immunostaining with antibodies against collagen-I (**E**–**F**) reveal structural changes in the inner and outer nuclear layers as well as collagen I expression in the outer retina of JR5558 mice, compared to naïve C57Bl/6J controls. Scale bars = 50 μm.

We next histologically assessed the retinal changes in JR5558 mice. Paraffin embedded eyes were sectioned and stained with Hematoxylin and Eosin (H&E) as well as Picro–Sirius Red (PSR) to assess structural alteration within the neural retina and extra cellular matrix alterations throughout the posterior eye cup. JR5558 mice had obvious structural changes within the neural retina with penetrating neovascularisation and disruption from the inner nuclear layer to the retinal pigment epithelium (RPE) (Fig. 2A–D). Increased extracellular collagen indicating a fibrovascular component was noted in areas where the retina had clear structural changes and lesions through the RPE (Fig. 2C–D). Immunostaining of frozen sections confirmed increased collagen-I in subretinal tissues (Fig. 2E–F).

**Figure 3:**
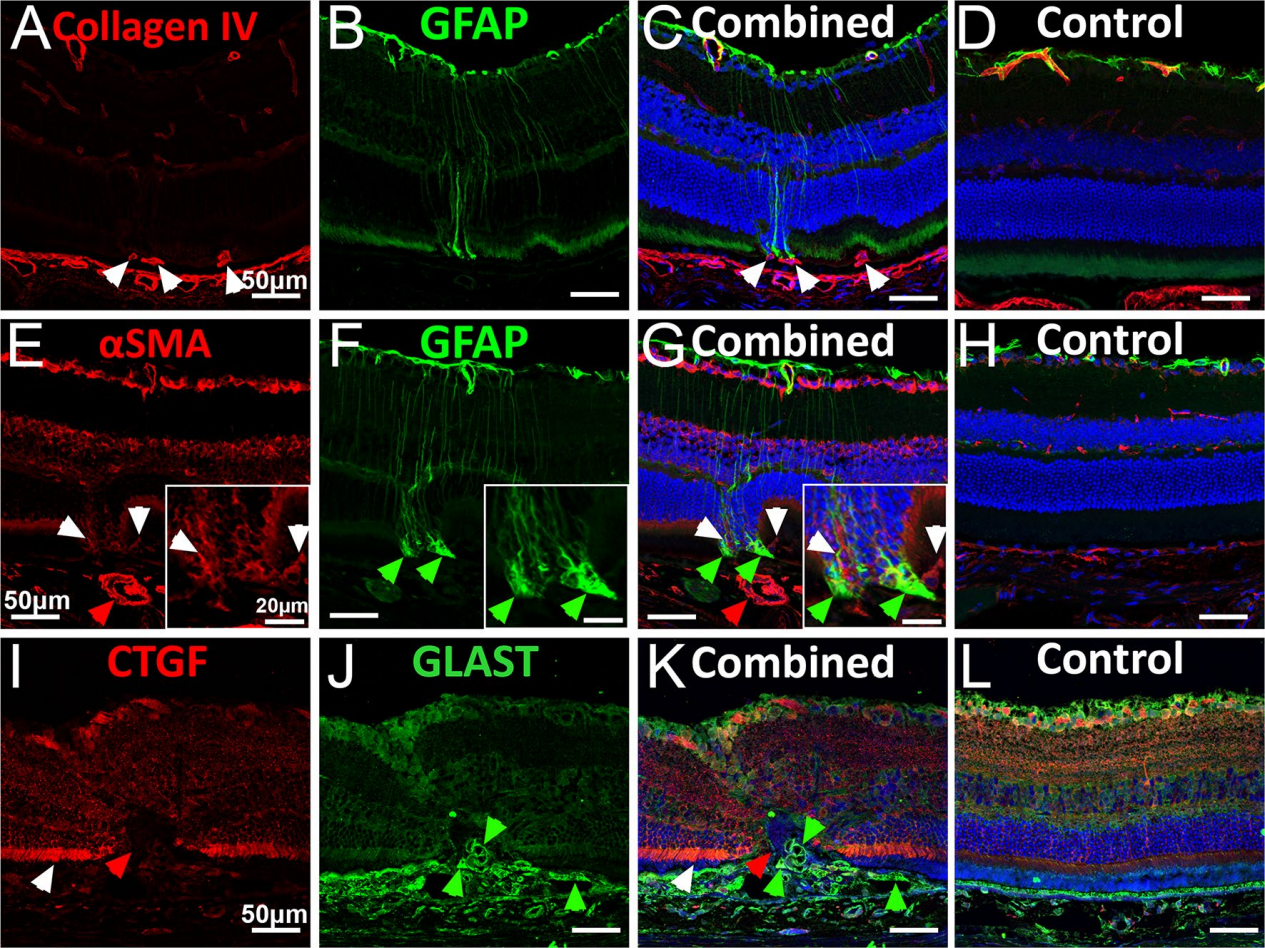
Extracellular matrix, ⍺SMA, GLAST and Müller glia marker expression in JR5558 mice. Immunofluorescence imaging of JR5558 retinas with antibodies against collagen-IV, ⍺-smooth muscle actin (⍺SMA), and glial fibrillary acidic protein (GFAP) reveal collagen IV (A–D, white arrowheads) and ⍺SMA expression (E–H, white arrowheads, magnified in insert) aligning with subretinal Müller cell gliosis (green arrowheads) in fibrovascular lesion areas. Immunofluorescence imaging JR5558 retinal sections with antibodies against connective tissue growth factor (CTGF) and glutamate-aspartate transporter (GLAST) reveal altered CTGF expression (red) and GLAST expression in the outer retina (I–K, red arrowhead: area lacking CTGF expression, white arrowhead: CTGF expression, green arrows: GLAST expression.), compared to compared to naïve C57Bl/6J controls (L). Panels A–C, E–G and I–K are images from JR5558 retinal sections, where panels C, G and K are composites. Panels D, H, and L are composites of retinal sections from age matched naïve C57Bl/6J mice. Inserts represent a magnification within the corresponding panels. Scale bars = 50 μm, inserts = 20 μm.

Additionally, expression of collagen-IV was noted in subretinal tissues (Fig. 3A,C) in areas with altered retinal structure, either with or without gliosis (Fig. 3A–C), as indicated by co-labelling of Müller cell marker glial fibrillary acidic protein (GFAP) in JR5558 but not in control mice (Fig. 3D). Similarly, staining of ⍺-SMA was found in the outer retina and subretinal space of JR5558 mice (Fig. 3E, magnified in insert, red and white arrowheads highlight areas of expression), but not in naïve C57BL/6J controls (Fig. 3H). Co-labelling with GFAP revealed Müller cell gliosis in JR5558 retinas (Fig. 3F,G) aligned with areas of ⍺-SMA positive staining (Fig. 3E–G, magnified in insert, green arrowheads highlight gliosis, white arrows highlight close ⍺-SMA expression) and neovessels (Fig. 3E,G, red arrow) in the subretinal space. Connective tissue growth factor (CTGF) is a signalling and regulatory molecule involved in proliferation, wound healing and angiogenesis, as well as tumour development and fibrosis^27^. We found that CTGF was highly expressed in the outer retina of JR5558 mice, compared to C57BL/6J controls (Fig. 3I,K,L; white arrow indicating CTGF expression, red arrow highlighting absence of expression). Staining with an antibody against glutamate-aspartate transporter (l‐glutamate‐l‐aspartate, GLAST) revealed altered distribution of GLAST expression in the subretinal space of JR5558 mice compared with C57BL/6J control retinas (Fig. 3J–L, green arrows highlight GLAST expression).

**Figure 4:**
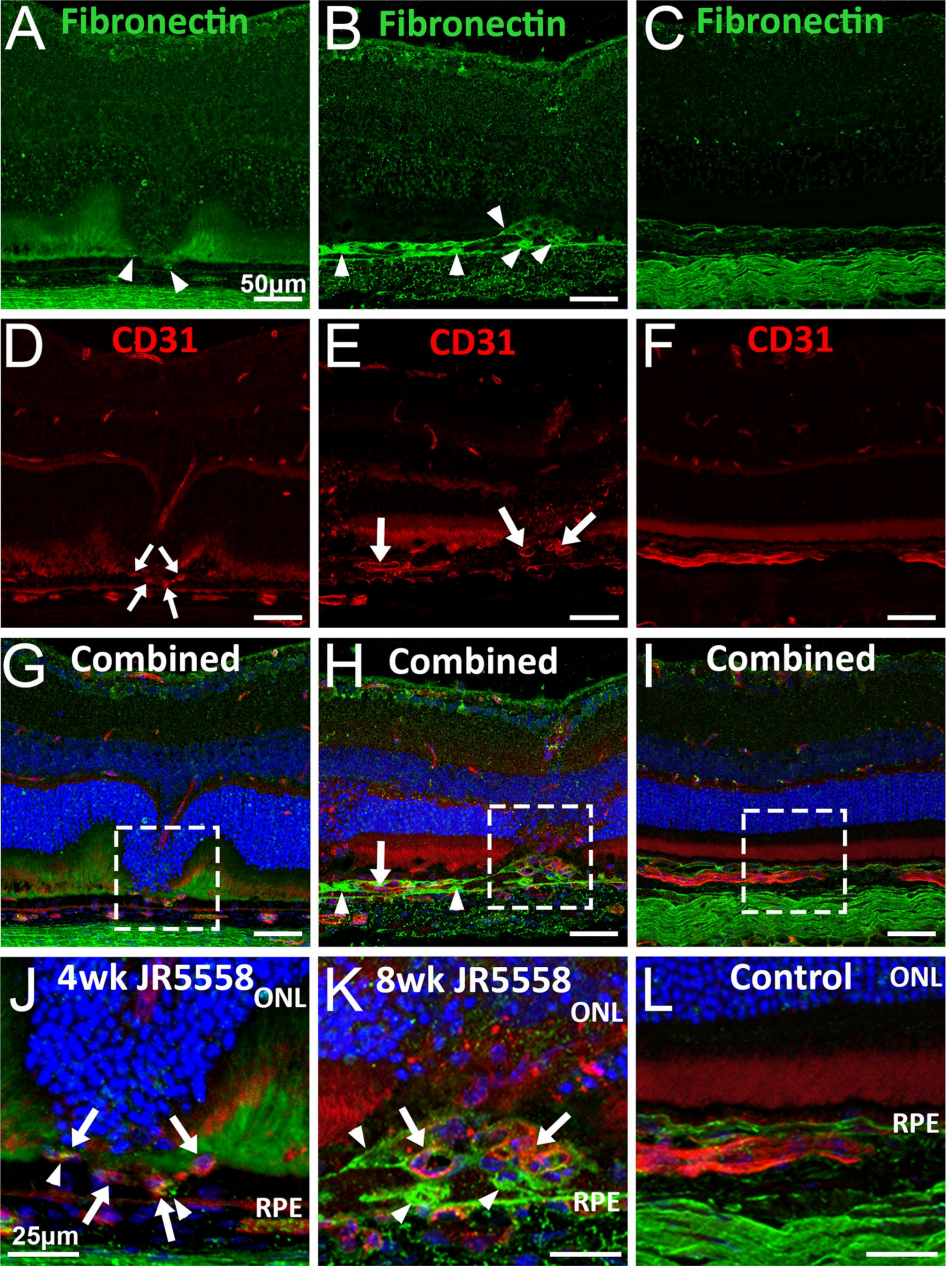
Fibronectin expression localise around neovessels in the subretinal space of JR5558 mice. Immunofluorescence imaging reveals fibronectin expression localised around CD31 stained neovessels, and increased over time, in the fibrovascular lesion areas of retinal sections from JR5558 mice compared to naïve C57Bl/6J control retinas. Panels A, D, G (composite) and J (magnification of panel G) are of 4-week-old JR5558 retinal sections, whilst panels B, E, H (composite) and K (magnification of panel G) are of 8-week-old retinal sections from JR5558 mice. Panels C, F, I (composite) and K (magnification of panel G) are stained retinal sections from age matched naïve C57Bl/6J mice. The outer retina and RPE area (dashed square G–I) are magnified in panels J-L. Arrowheads indicate areas of distinct fibronectin expression. Arrows indicate neovessels within the outer retina. Scale bars: A–I = 50 μm, J–L = 20 μm.

We next investigated fibronectin expression in JR5558 eye cups. Altered fibronectin expression could be noted at 4 weeks in JR5558 mice and varied between notable signal within the photoreceptor layer to areas of distinct fibronectin staining localised to areas with the outer nuclear layer subsiding into the photoreceptor layer (Fig. 4A, arrowheads). Analysis of 8-week JR5558 retinas revealed significant distinct fibronectin staining in the subretinal space, with clear fibronectin expression in lesions areas (Fig. 4B, arrowheads). No fibronectin staining was noted in the neural retina of naïve C57BL/6J controls (Fig. 4C, magnified in 4L). Staining for CD31 revealed the presence of neovascularisation in the subretinal area at 4 and 8 weeks in JR5558 mice (Fig. 4D–E, arrows), but not present in C57BL/6J controls (Fig. 4F). Subretinal neovascularisation was observed in areas with increased fibronectin (Fig. 4G–H, the outer retina and RPE area magnified in Fig. 4J–L, arrowheads indicate areas of distinct fibronectin expression and arrows indicate neovessels) in JR5558 mice but not in C57BL/6J controls (Fig. 4I,L).

**Figure 5:**
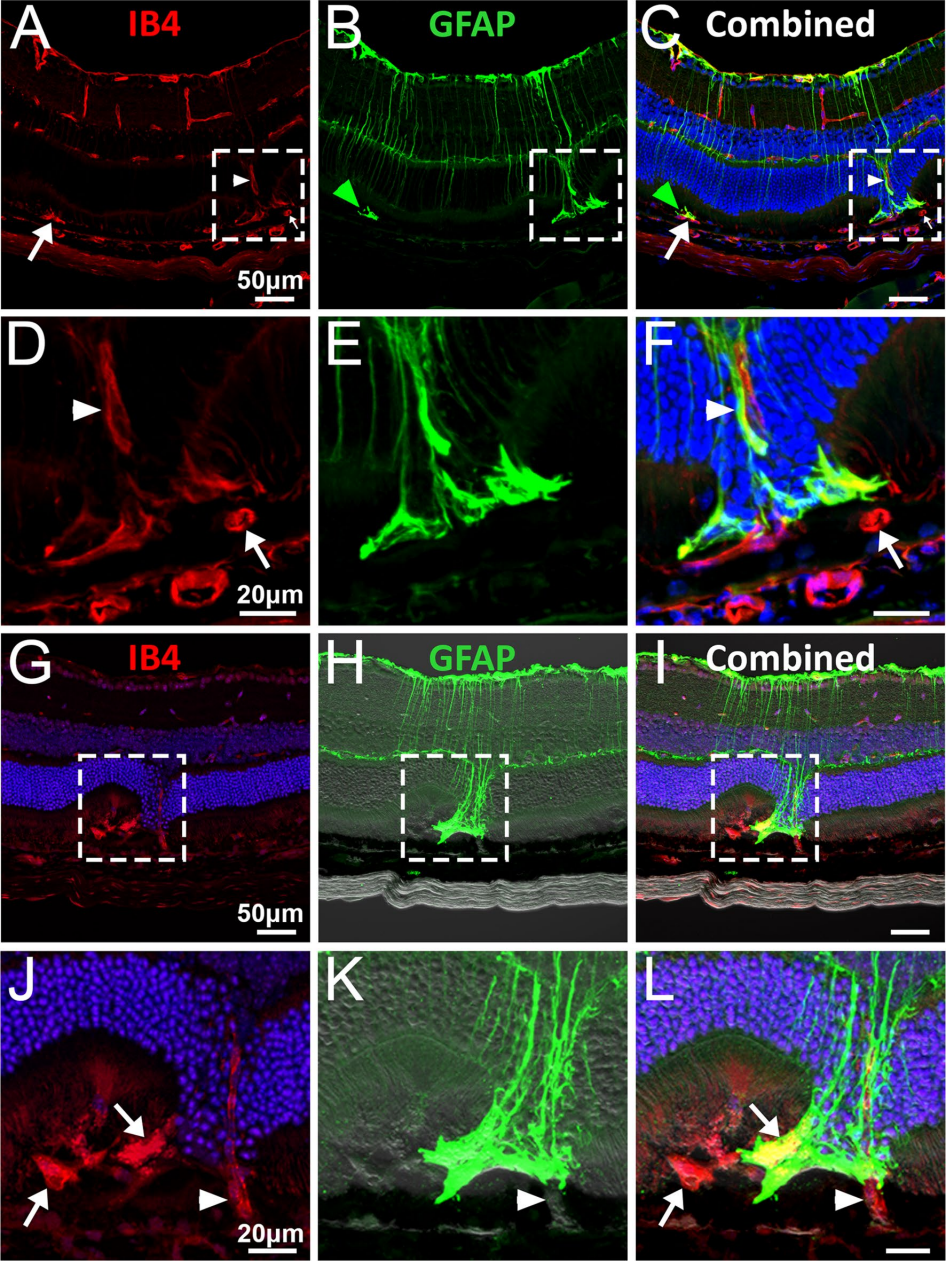
Penetrating neovessels in JR5558 retinas originate from both the retinal vascular plexus and choroid. Immunofluorescence imaging of outer retinal neovascularisation and gliosis in JR5558 mice reveal examples of neovessels originating from retinal vascular plexus are presented in (panels D–F represent magnification of panel A–C, arrowhead) as well as neovessels of choroidal origin that penetrate the RPE (panels J–L represent magnification of panel G–I, arrowhead). Subretinal vessels perpendicular to the section plane were noted in subretinal lesion areas of JR5558 sections (arrows). Panels H–I & K–L have bright-field image added to indicate pigmented cells. Scale bars: A–C & G–I = 50 μm; D–F & J–L 20 μm.

We next sought to further assess neovascularisation and gliosis in the outer retina of JR5558 mice. Neovessels originating from the outer-plexiform layer and extending through the outer nuclear layer towards the subretinal space (Fig. 5A,D, arrowhead; dotted box in panels A–C is magnified in D–F) and ending at the subretinal layer were noted in JR5558 mice (Fig. 5A–F). Gliotic cells ran parallel along these vessels (Fig. 5B) and extended into the fibrovascular subretinal area (Fig. 5E, green arrowhead highlighting another example in panels B–C). Subretinal neovessels perpendicular to the section plane were noted in some subretinal areas of JR5558 sections (arrows in Fig. 5A–F). Neovessels that penetrate the RPE at areas of subretinal gliosis were also noted in some JR5558 eyes (Fig. 5G–I, dotted box magnified in J–L, arrowhead indicating location of RPE penetrance noted by overlay with bright-field image). Additional examples of subretinal vessels perpendicular to the section plane were noted in this area (Fig. 5J,L, arrows).

Upregulation of intermediate filament proteins GFAP and vimentin, cell proliferation and cell growth in glial cells are hallmarks of gliosis^28,29^. Co-staining retinal sections with vimentin and cellular retinaldehyde–binding protein (CRALBP), a retinoid-binding protein expressed in RPE and Müller cells^30^ revealed reactivated Müller glia processes, double-positive for vimentin and CRALBP, expanding into the subretinal space (Supplemental Fig. 2A–C, arrows in dotted area magnified in D-F). Altered CRALBP expression was also noted around the area with subretinal lesions, with increased CRALBP expression in RPE away from the subretinal lesion (Supplemental Fig. 2B–C, arrowheads in B) and reduced CRALBP expression and altered RPE structure noted around subretinal lesion (Supplemental Fig. 2E–F, arrowheads in E). Reactivated Müller glia processes and altered RPE structure were not seen in C57BL/6J controls (Supplemental Fig. 2G–I).

Taken together, these findings indicate that fibrovascular subretinal lesions have increased α-SMA and GLAST expression, increased collagen-I, collagen-IV and fibronectin deposits, gliosis, and are vascularised by neovessels of either choroidal or vascular plexus origin. Further, CTGF expression was increased in the outer retina of JR5558 mice.

### JR5558 mice develop altered RPE and photoreceptors

**Figure 6:**
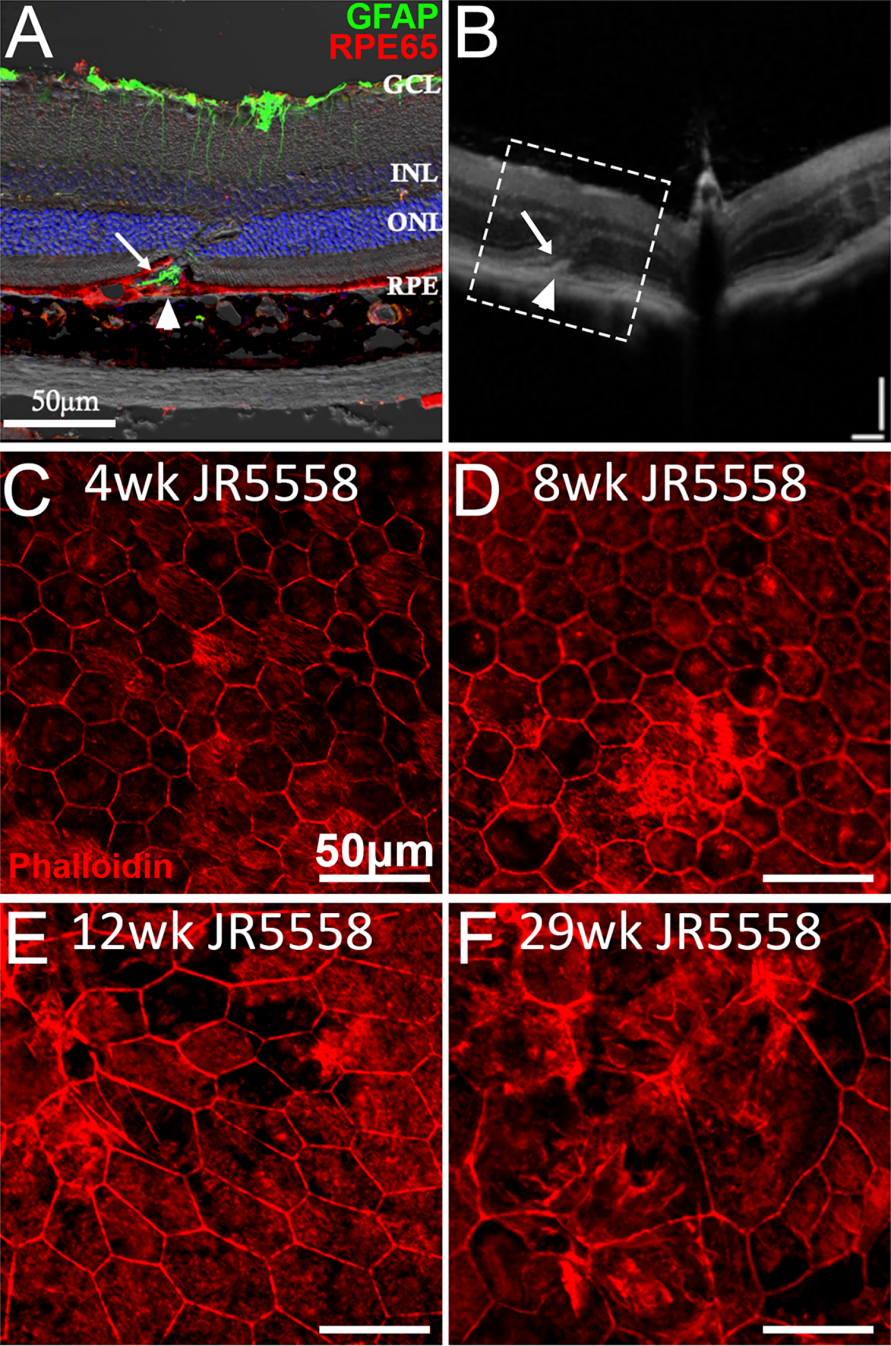
Altered RPE structure and cell morphology in JR5558 mice. Immunofluorescence imaging of JR5558 retinal sections with antibodies against GFAP (green) and RPE65 (red) revealed subretinal Müller cell gliosis and altered RPE structure around the lesion area (**A**), corresponding to the altered outer retina and RPE structure noted in OCT image (**B**). Arrow and arrowhead highlighting subretinal gliosis and altered RPE, respectively. Phalloidin staining of JR5558 retinal flatmounts revealed the normal morphology of RPE cells at 4 (**C**) and 8 weeks (**D**) is changed from 12 weeks onwards (**E**–**F**). Dashed box represents area imaged in panel A. Scale bars: 50 μm.

Co-labelling retinal sections for GFAP and the RPE specific marker RPE65 indicated that subretinal Müller cell gliosis was accompanied by alterations of RPE cell structure (Fig. 6A), which was clearly visible in OCT images (Fig. 6B, dotted area represents area imaged in 6A, arrow and arrowhead highlighting subretinal gliosis and altered RPE in A-B, respectively). Phalloidin flatmount staining of the RPE flatmounts from JR5558 mice revealed relatively less disrupted RPE morphology and density at 4 and 8 weeks (Fig. 6C–D) but RPE disruption was obvious from 12 weeks of age onwards (Fig. 6E–F).

**Figure 7:**
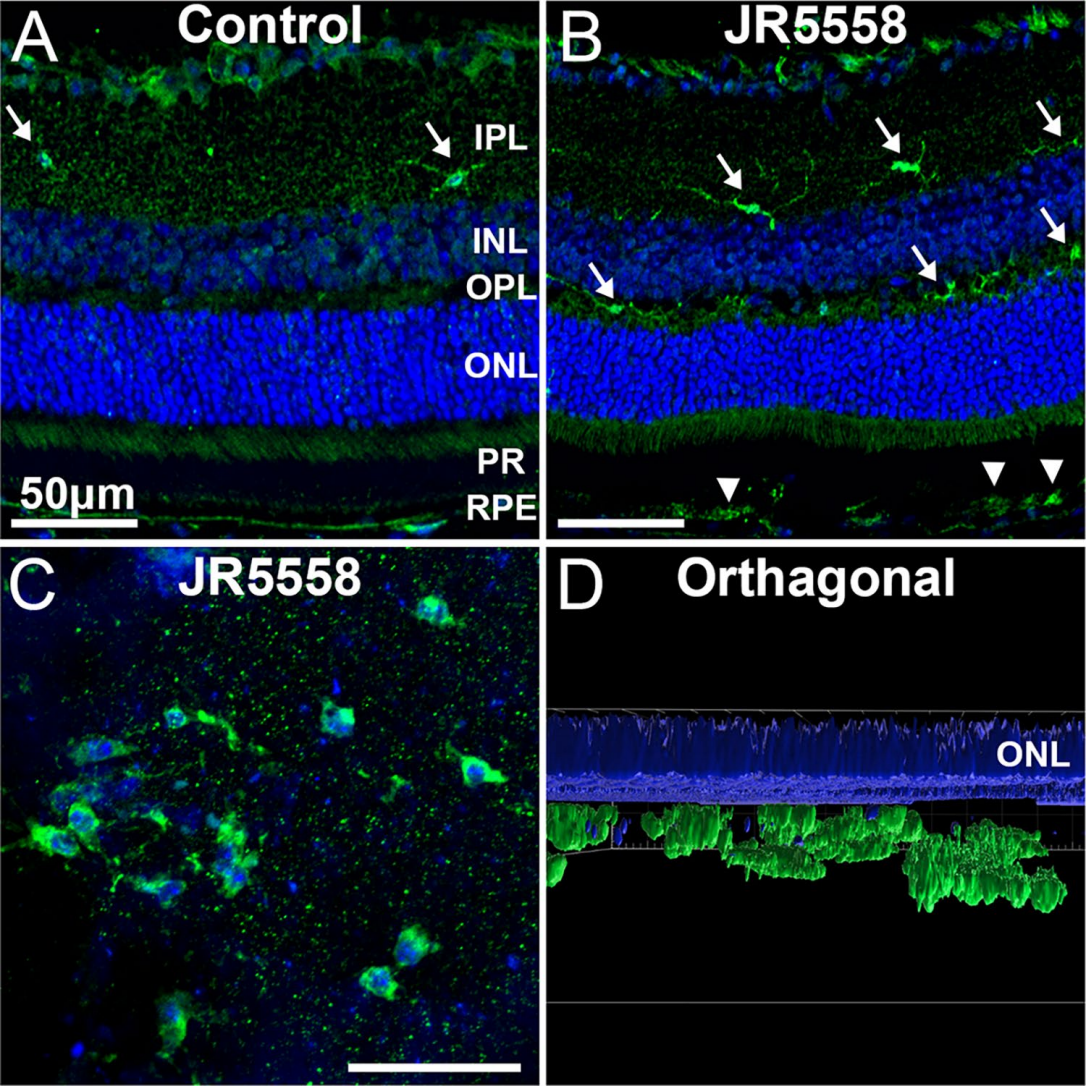
Increased microglia distribution in the outer retina of JR5558 mice. Immunofluorescence imaging of JR5558 retinal sections with antibodies against Iba-1 reveal increased ramified microglia in the inner and outer plexiform layers, as well as amoeboid microglia in the outer retain, compared to naïve C57Bl/6J controls (**A**–**B**). Flatmount staining of JR5558 eye cups confirmed microglia in the outer retina (**C**), located sub-ONL (**D**). Panel D is the orthagonal view of panel C, rendered for clarity. Scale bars: 50 μm.

We next investigated alterations in microglia to reflect changes in the resident phagocytic and supportive immune reactions within the retina in JR5558 mice. Under steady state conditions, Ionized calcium binding adaptor molecule (Iba)-1 positive microglia populated the inner plexiform layer of the neural retina (Fig. 7A, arrows). Analysis of JR5558 retinas revealed increased number of ramified Iba-1 in the inner and outer plexiform layers of the retina (Fig. 7B, arrows), along with amoeboid microglia infiltrating into the subretinal space (Fig. 7B, arrowheads). Analysis of JR5558 retinal flatmounts confirmed the presence of Iba-1 positive microglia in the outer retina (Fig. 7C, arrows), located sub-ONL (Fig. 7D, orthagonal view of 7C, rendered for clarity) (Supplemental Video 1).

As subretinal gliosis, altered RPE, and fibrovascular lesions can all affect the health of photoreceptors, we next studied changes in photoreceptors by staining retinal sections with antibodies against cone arrestin, which stains cone photoreceptors including outer segments and synaptic bodies, and rhodopsin, which stains the outer segments of rod photoreceptors. The highly organized and uniformly distributed photoreceptor morphology in steady state naïve retina (Supplemental Fig. 3A–C) was disrupted with focal disorganisation of the outer retinal layers (Supplemental Fig. 3D–F, and J) as well as areas of rod and cone loss (Supplemental Fig. 3G–J) in JR5558 retinas.

### Extracellular matrix and fibrotic markers quantifiably increase in JR5558 mice

**Figure 8:**
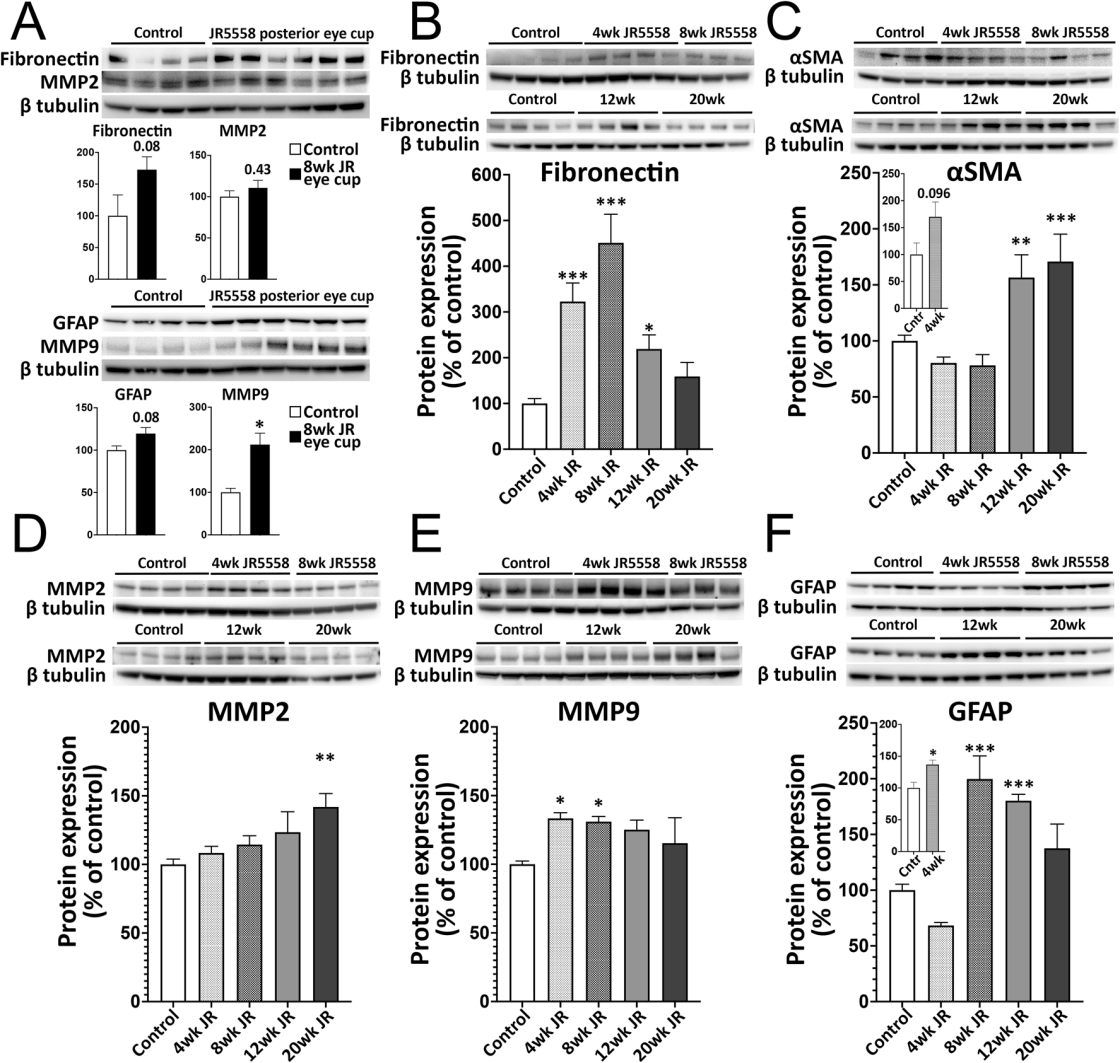
JR5558 retinas have altered protein expression in extracellular matrix and fibrotic markers. Western blot analysis of whole posterior eye cups revealed significant or near significant increases in fibronectin (*p* = 0.080), GFAP (*p* = 0.078), and MMP9 (*p* = 0.012) in JR5558 mice compared to 9-week-old naïve C57Bl/6J controls (**A**). Western blot analysis of lysed neural retinas revealed increased fibronectin expression at 4- (*p* < 0.001), 8- (*p* < 0.001) and 12-week (*p* = 0.025) JR5558 mice (**B**), whereas α-SMA expression significantly increased later at 12- (*p* = 0.009) and 20-weeks (*p* < 0.001) (**C**). Insert: α-SMA expression of neural retinas from 4-week-old JR5558 mice compared to 4-week-old naïve C57Bl/6J retinas. MMP-2 expression increased in the neural retina to reach significance at 20 weeks (*p* = 0.001) (**D**), whereas MMP-9 expression was elevated at 4- (*p* = 0.011), 8- (*p* = 0.043), 12- (*p* = 0.082), and 20-week-old (*p* = 0.46) (E). GFAP expression elevated at 8- (*p* < 0.001), 12- (*p* < 0.001), and 20-week-old (*p* = 0.15) animals (Fig. 8F). Insert: GFAP expression at 4-weeks compared to 4-week-old naïve C57BL/6J retinas (*p* = 0.014). Panels A, C insert and F insert: t-test, *p < 0.05; Panels B–F: One-way ANOVA, *p < 0.05, **p < 0.01, ***p < 0.001.

To determine the timeline of ECM and fibrosis markers, western blots were performed with lysed retinas of JR5558 mice and compared to 9-week-old naïve C57BL/6J controls. Initially, protein levels of whole posterior eye cups were compared and significant or near significant increase in fibronectin (172.7 ± 20.2 vs. 100 ± 32.9, *p* = 0.080, t-test), GFAP (119.5 ± 7.0 vs. 100 ± 5.0, *p* = 0.078), MMP2 (110.6 ± 9.2 vs. 100 ± 7.1, *p* = 0.429) and MMP9 (212.1 ± 26.8 vs. 100 ± 9.5, *p* = 0.012) were noted in JR5558 mice (Fig. 8A). To dissect protein changes specific to the retina, WB analysis of neural retinas from control and JR5558 mice were next performed. Fibronectin expression increased over threefold (322.8 ± 40.4% vs. 100 ± 10.8, *p* < 0.001, ANOVA), 4.5-fold (451.0 ± 62.7, *p* < 0.001) and twofold (219.0 ± 31.0, *p* = 0.025) at 4, 8 and 12 weeks, respectively (Fig. 8B). Whilst fibronectin expression was elevated at 20 weeks, this increase was not significant (159.1 ± 30.7%, *p* = 0.47). α-SMA expression was significantly elevated at 12 (156.4 ± 20.3% vs. 100 ± 5.1, *p* = 0.009) and 20 (170.8 ± 24.5, *p* < 0.001) week old JR5558 mice (Fig. 8C). α-SMA expression also increased at the earlier timepoint of 4 weeks (170.1 ± 27.5% vs. 100 ± 21.5, *p* = 0.096, t-test) when the neural retinas of 4-week-old JR5558 mice to 4-week-old naïve C57BL/6J retinas (Fig. 8C, insert). Matrix metalloproteinase (MMP)-2 and MMP-9 play a significant role in the etiology of AMD^31,32^. We found MMP2 expression to steadily increase over time in the neural retina of JR5558 mice (4 wk: 108.4 ± 4.8%, *p* = 0.87; 8 wk: 114.5 ± 6.4, *p* = 0.54; 12 wk: 123.5 ± 14.9, *p* = 0.11), with increases only becoming statistically significant at 20 weeks (141.8 ± 9.8% vs. 100.0 ± 3.9, *p* = 0.001) (Fig. 8D). The expression of MMP-9 was elevated in 4- (133.4 ± 4.1% vs. 100 ± 2.3, *p* = 0.011), 8- (131.0 ± 3.8, *p* = 0.043), 12- (125.1 ± 7.0, *p* = 0.082), and 20-week-old (115.3 ± 18.5, *p* = 0.46) JR5558 neural retinas, compared to C57BL/6J controls (Fig. 8E). WB analysis of JR5558 neural retinas was also consistent with our immunohistochemical staining results, with GFAP expression elevated at 8- (199.7 ± 02.8% vs. 100 ± 5.4, *p* < 0.001), 12- (180.3 ± 5.7, *p* < 0.001), and 20-week-old (137.6 ± 21.8, *p* = 0.15) animals (Fig. 8F). As GFAP expression at the earlier 4-week timepoint was less than that of the 9-week-old C57BL/6J controls, we further assessed the neural retina in 4 week old animals and found GFAP expression was increased in 4-week old JR5558 mice were compared to 4-week-old naïve C57BL/6J retinas (136.8 ± 7.1% vs. 100 ± 9.0, *p* = 0.014, t-test) (Fig. 8F, insert).

We also assessed protein changes in the RPE/choroid complex, as subretinal fibrovascular lesions may adhere to this complex when the neural retina is separated. Fibronectin expression increased over twofold (225.9 ± 85.8% vs. 100 ± 24.2, *p* = 0.13) at 4-, and over fivefold (555.9 ± 58.2, *p* < 0.001) at 8-weeks in JR5558, compared to controls. No change was noted in 12- (88.5 ± 8.2, *p* = 0.99) and 20-week-old JR5558 mice (75.6 ± 4.3, *p* = 0.99) (Supplemental Fig. 4A). A significant increase in α-SMA expression was noted in the RPE/choroid complex of 8-week-old JR5558 mice (163.0 ± 11.6% vs. 100 ± 3.7, *p* < 0.001) (Supplemental Fig. 4B). While expression of MMP-2 followed a similar trend to that of the neural retina, steadily increasing up to 12 weeks (4 wk: 111.6 ± 17.2% vs. 100 ± 8.5, *p* = 0.88; 8 wk: 134.5 ± 9.1, *p* = 0.11; 12 wk: 144.9 ± 8.5, *p* = 0.01) (Supplemental Fig. 4C), MMP-9 expression was variable, significantly decreasing in the RPE/choroid complex of 20-week-old JR5558 mice (156.4 ± 20.3 vs. 100 ± 5.1, *p* = 0.009) (Supplemental Fig. 4D).

### Lesions in fundus and OCT align with gliosis in immunohistochemical stained sections and retinal flatmounts

**Figure 9:**
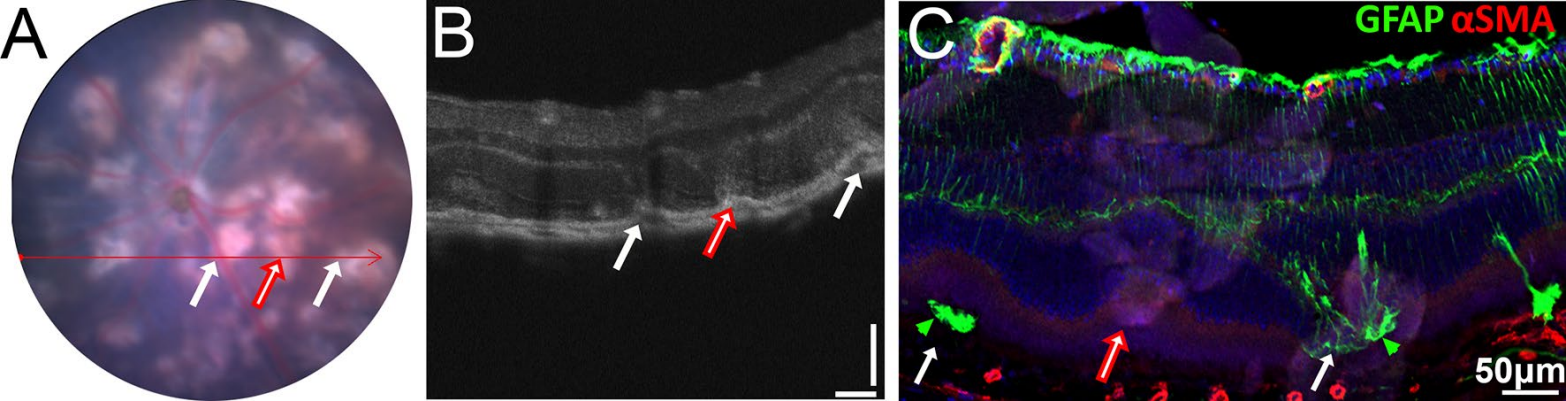
Lesions in fundus and OCT align with gliosis in immunohistochemical stained sections and retinal flatmounts Brightly yellow subretinal lesions noted in fundus (**A**) and OCT images (**B**) aligned with subretinal areas of gliosis in fixed retinal sections of the same retina stained with antibodies against GFAP and α-SMA (also highlighting vasculature) (**C**). GFAP positive Müller cell gliosis (green arrowheads) was not present in the subretinal space of all subretinal lesions (Fig. 9A–C, middle red arrow highlights subretinal lesion lacking gliosis). Scale bars: A = 200 μm, B = 100 μm and C = 50 μm.

We next sought to confirm lesions noted in fundus and OCT images aligned with areas of subretinal gliosis in retinal sections. Utilising GFAP as a marker for retinal fibrosis and retinal vasculature for alignment, retinal sections and flatmounts of JR5558 mice beyond 8 weeks of age were stained and compared with fundus and OCT images. Brightly yellow subretinal lesions in fundus images, as well as the associated OCT images, were compared to retinal sections from the same animal, and areas of subretinal fibrosis found to align with areas of gliosis demonstrated immunohistochemically (Fig. 9A–C) and, in some cases, subretinal neovascularisation (Fig. 9C). Interestingly, not all subretinal lesions in fundus and OCT images had GFAP positive Müller cell gliosis in the subretinal space (Fig. 9A–C, middle red arrow).

**Figure 10:**
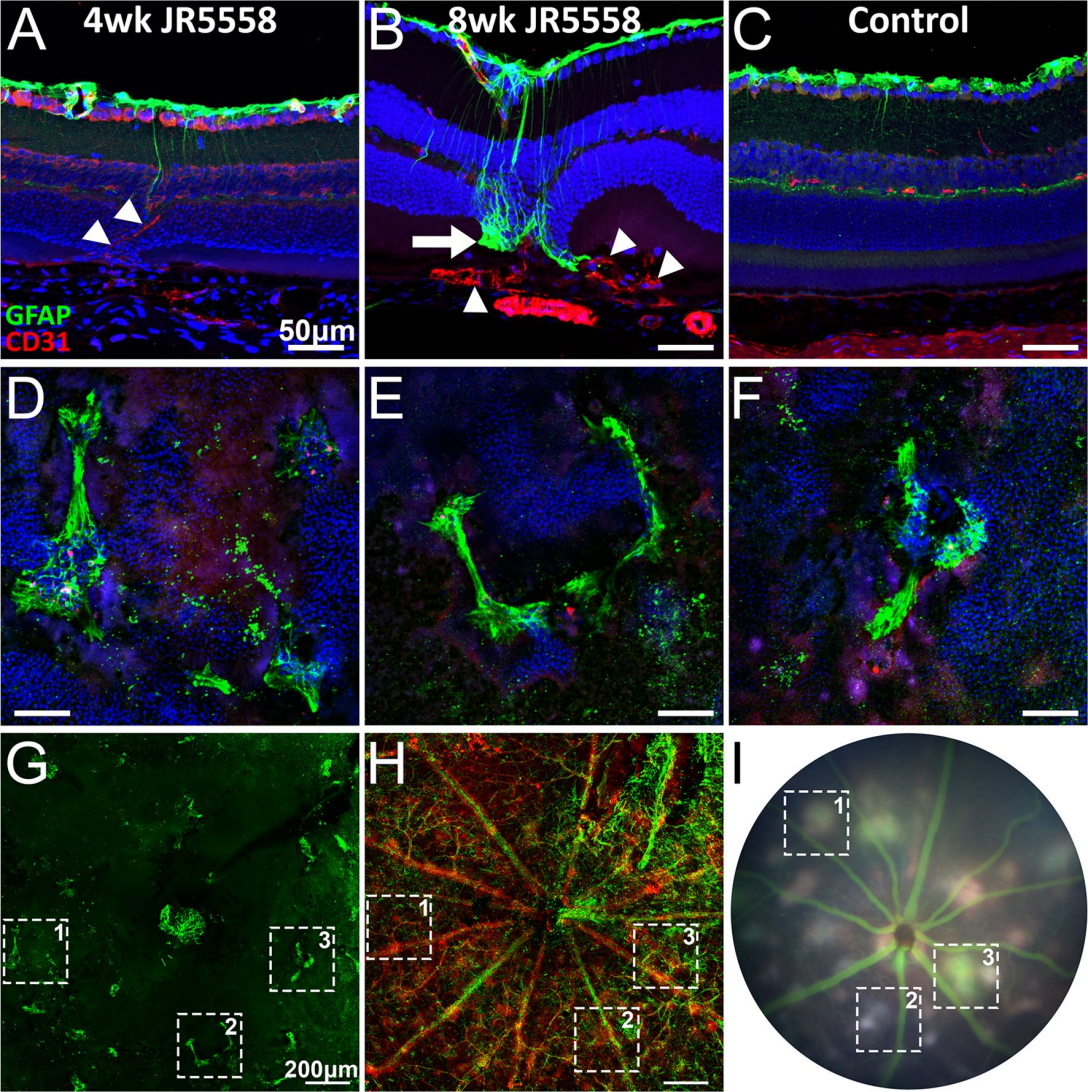
Subretinal gliosis in retinal flatmounts align with retinal OCT images. Immunofluorescence imaging of JR5558 retinal sections with antibodies against GFAP (green) and CD31 (red) revealed neovessels (arrowheads) but not gliosis (arrow) at 4 weeks (**A**), whereas both gliosis and neovessels are significant at 8 week (**B**) and older JR5558 retinas compared to 4wk JR5558 and naïve C57BL/6J controls (**C**). Gliosis in the photoreceptor layer (sub-ONL) of retinal flatmounts stained with antibodies against GFAP (green, **D**–**G**). Utilising CD31-positive retinal vasculature as a guide, areas of subretinal gliosis were aligned with fundus images taken from the same animal (**G**–**I**). Dashed boxes represent the same area across panels. Numbered areas 1–3 represent the same area in panels G–H, and coincide with panels D–F, respectively. Scale bars: A–F = 50 μm, G–H = 200 μm.

Despite some variability in the onset of fibrotic lesion in JR5558 mice, gliosis at the subretinal space was consistently noted in 8-week-old and older JR5558 retinas, compared to 4-week-old JR5558 and control mice (Fig. 10A–C, arrowheads indicating neovessels, arrow indicating subretinal gliosis). Retinal flatmounts stained with antibodies against GFAP confirmed the presence of gliosis in the photoreceptor layer (sub-ONL) (Fig. 10D–G). Imaging through the retinal flatmounts, and utilising CD31 positive retinal vasculature, areas of subretinal gliosis were aligned with fundus images taken from the same animal (Fig. 10G–I, dotted boxes represent the same area, and coincide with panels D–F).

By comparison, subretinal gliosis (Supplemental Fig. 5A,B, green arrowhead), as well as α-SMA positive staining in the lesion area (Supplemental Fig. 5B, arrowhead), was noted in some 4-week-old JR5558 mice. Analysis of retinal flatmounts of 4-week-old mice revealed the presence of smaller subretinal clusters of gliosis (Supplemental Fig. 5C). Imaging through the retina, with CD31 positive retinal vasculature as a guide, areas of gliosis were aligned with lesion areas on fundus images taken from the same animal.

### Analysis of subretinal hyperreflective fibrotic lesion following intravitreal treatment

**Figure 11:**
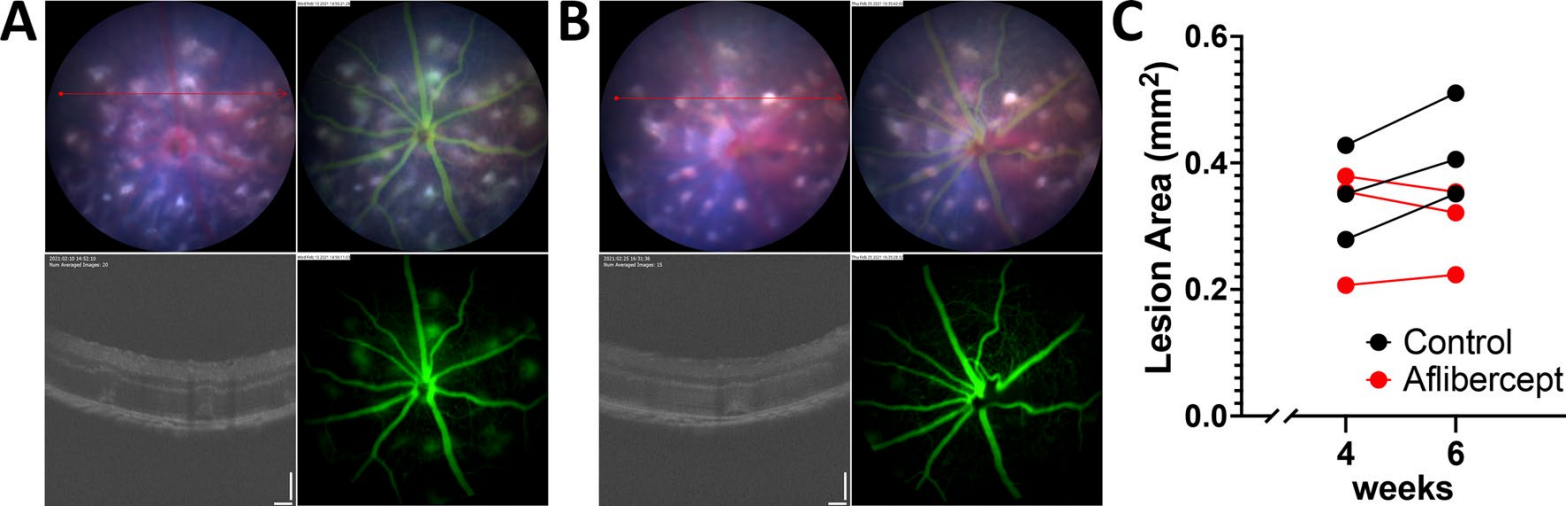
Vascular leak and lesion area quantification following intravitreal Aflibercept treatment. Fundus photography, OCT imaging and fluorescein-angiography on JR5558 mice before (**A**) and 2 weeks following intravitreal injection of Aflibercept (**B**) reveal a reduction in vascular leak and subretinal lesions, with no growth in lesion area observed compared to untreated controls (**C**) (n = 4 per group).

Vascular endothelial growth factor (VEGF) inhibitor, Aflibercept, is the gold-standard treatment for neovascular AMD, decrease vascular leakage and haemorrhage to improve vision acuity. We performed intravitreal Aflibercept injection treatment into 4-week-old JR5558 mice and assessed vascular leak and lesion area 2-weeks following anti-VEGF treatment. Vascular leak noted at the 4-week timepoint were not observed post-treatment (at 6 weeks, Fig. 11A,B), and OCT images revealed reduction in abnormality of the outer retinal segment as well as reduced lesion growth in fundus photographs (pretreatment at (4 weeks): 0.31 ± 0.05 mm^2^, range: 0.21–0.37; post-treatment (6 weeks): 0.30 ± 0.04, range: 0.22–0.35, *p* = 0.47, n = 3), compared to sham controls (4 weeks: 0.35 ± 0.04, range: 0.28–0.43; 6 week: 0.42 ± 0.05, range: 0.35–0.51, *p* = 0.01, n = 3). This represents an average 3% decrease in the treatment group compared to an average 20% increase in controls.

## Discussion

Retinal fibrosis is characterized by excessive deposition of collagenous extracellular matrix (ECM) proteins by various cell populations, including Müller glia, microglia, and the retinal pigment epithelium (RPE)^8–10^. Herein we systematically characterise the development of subretinal fibrovascular lesions and changes in the expression of ECM proteins in the retina of the JR5558 mice. Our study indicates that JR5558 mice are a reliable model to investigate subretinal fibrosis due to its consistent development of numerous subretinal fibrovascular lesions that are accompanied by subretinal gliosis, disrupted RPE cells and photoreceptors injury. We also provide proof of concept that these mice can be used to assess the efficacy of intravitreal treatments/ interventions.

The JR5558 mice were found to exhibit a unique neovascular phenotype created through the Jackson Laboratory Eye Mutant Screening program^33^. These mice carry autosomal recessive mutations in Crb1^rd8^ and *Jak3*^*m1J*^. While mutation in the *Crb1*^*rd8*^ allele is necessary for the retinal vascular disease phenotype, including disturbance of the outer retina and decline in scotopic and photopic responses to visual stimuli, mutation in both this and the *Jak3*^*m1J*^ allele are required for the seen in the JR5558 mice^34^. To date, several publications have utilised this line for its spontaneous choroidal neovascularization (CNV) and features of retinal degeneration^24,25,34–44^. A previous study indicates that neovascular lesions in JR5558 mice are characterised by persistent vascular leak, resulting in retinal oedema, focal gliosis and photoreceptor damage^24^. Our results confirm the occurrence of spontaneous CNV and highlight instances of abnormal blood vessels across the retina, including the outer plexiform layer, outer nuclear layer, penetrating the photoreceptor cell layer and further extending into the subretinal space. The latter was previously described by Hasegawa et al.^25^ and Nagai et al.^24^, with both studies confirmed neovessels to be of choroidal origin. Pigmentary changes in CFP were also noted in both studies.

Nagai et al. noted multifocal pigmentary changes, while Hasegawa et al. described “multiple areas of retinal depigmentation … in the posterior fundus” of JR5558 mice^24,25^. Melanin in the eye within the choroid, iris, and retinal pigment epithelium (RPE) is responsible for the absorption of excess light radiation from the photoreceptors, scavenging light-generated oxygen reactive species and reactive oxygen species^45,46^. Whilst changes in RPE pigmentation are seen with normal aging, it also occurs in diseases such as albinism and age-related macular degeneration^47^. We found structural and morphological alterations in the RPE but found no RPE depigmentation at any of the timepoints or JR5558 mice assessed. Interestingly, analysis of the retinal sections presented within the original study by Hasegawa et al.^25^ reveals the RPE layer retaining pigmentation, further indicating the lesions area in CFP are not the result of pigmentary loss in JR5558 mice.

Spontaneous subretinal neovascular lesions have been tracked and quantified over time at both baseline and following targeted treatments using the JR5558 mouse line^24,36–41^. Lesions in these studies refers to choroidal neovessels penetrating through the RPE. Whilst VEGF inhibitors are effective antiangiogenic agents that can help resolve leakage from CNV, the subretinal fibrotic scarring in eyes with nAMD persist despite receiving this treatment^3^. We found yellow lesion-like areas of the fundus consistently emerge and expand from 4 weeks. Aside from the large variation in lesion formation between 3- and 4-week timepoints, representative images between 4 and 20 weeks of the JR5558 fundus are presented in this study. Similar fundus alterations, with focal areas occurring at day 21, correlating with the development of vascular leakage which peaked at day 25 and largely subsided by day 31, have been previously described^25^. This study (Hasegawa et al.), however, focused between postnatal day 17 and day 35 and did not quantify fundus changes after that. OCT images recorded in our study revealed the lesions are beneath the neural retina, and fluorescein angiography revealed areas of significant vascular leak leading up to 12 weeks and reducing beyond this timepoint. Whilst a similar pattern was noted in the literature, the timing of this reduction was different to our study. The vascular lesions were reported to became diffuse and enlarged with vascular leak reported to subside around day 31^25^, whereas we found lesions and vascular leak to be present even at 20 weeks. We next applied a modified semi-automated process, a technique previously utilised to quantify cellular morphology^48–50^, to quantify the frequency and 2D area of lesions located within the peripapillary region thereby tracking and quantifying subretinal lesions reliably over time in CFP images. Whilst no difference was found in the number of lesions between 4 and 20-weeks, analysis of the lesion area revealed an increase during this period. Further, we found some lesions resolve over time while others become established (8 weeks and beyond), indicating that some fibrovascular lesions regress whereas others become established.

Whilst obvious solid white/yellow mounds in the CFP along with accumulations of subretinal hyperreflective material in the OCT indicate subretinal fibrosis^3,26,51–54^, neovascular tissue, blood, inflammatory reaction or fibrin may also be seen as subretinal hyperreflective material with OCT^55–58^. As it is not possible to differentiate between fibrosis and these other forms of tissue deposits using conventional OCT, techniques such as polarization-sensitive optical coherence tomography (exploiting the birefringence of fibrotic tissue) can be utilised to identify fibrosis in human eyes^59^. The ECM components of sub-foveal AMD fibrotic lesions are reported to include collagen types I and IV and fibronectin^60^. Collagen type I and IV have been shown to be reliant on a fibronectin matrix for ECM incorporation^61–63^. Further, CTGF expression is increased in basal deposits/drusen in human AMD maculas and induces fibronectin and MMP-2 production^64^. Similarly, the cellular and extracellular components of the lesions can be assessed to further investigate subretinal fibrosis in JR5558 mice. Accordingly, we studied collagen I, collagen IV, fibronectin as well as gliosis to confirm the fibrotic component of the lesions seen in JR5558 mice. We found significant upregulation of CTGF, fibronectin and collage I expression in the retina of JR5558 mice. Collagen IV and ⍺SMA staining was also noted in and around the fibrovascular lesions. In addition to spontaneous CNV and fundus changes, the retina of JR5558 mice had obvious changes to the structure of the normally distinct layers, and in turn altered retinal function which has been confirmed in previous reports^25,35^. Results of western blot analysis were consistent with our immunofluorescence results, as indicated by marked increase in the expression of fibronectin, ⍺SMA, MMP2, and MMP9. MMP2 and MMP9 have been reported to play a key role in the pathogenesis of AMD and may cooperatively be involved in the progressive growth of choroidal fibrovascular lesions in nAMD^65^.

Vascular leak was not observed in all lesions assessed in JR5558 mice. This could be due to the lack of vessels within the lesion, differences in the vasculature state (normal vessels that do not leak have yet to become abnormal), or due to variable flow rates. Optical coherence tomography angiography (OCTA) studies of subretinal fibrosis in AMD described different types of neovessels within fibrotic scars^66^. One study highlighted neovessels within 93.8% of eyes assessed, with the remaining 6.2% termed as either ‘dark’ lesions with the scar having ‘large flow voids’ consisting of diffuse lack of signal, or a ‘dark halo’ where a dark ring surrounding the neovascular network in the choriocapillaris segmentation^13^.

α-SMA cellular staining that may indicate the presence of myofibroblasts within lesion sites^67^ was noted in the JR5558 retinal lesions. Neither the retina or neovessels contain fibroblasts to become activated and form the myofibroblasts responsible for the excessive production of extracellular matrix proteins required for pathogenic subretinal fibrosis. Accordingly, these cells must either be recruited or differentiate from other cell types, which include RPE cells, glial cells, vascular pericytes, as well as infiltrating macrophages^68^.

RPE cells contribute to retinal fibrosis through undergoing epithelial to mesenchymal transition (EMT)^69^, a feature well described in the development of fibrosis in several tissues such as the lung and kidney. TGF-β, described as the master regulator of fibrosis, as well as MMPs from inflammatory cells and the RPE^9^, induce EMT through activating downstream pathways including mitogen-activated protein kinases (MAPKs), SMAD2, phosphoinositide 3-kinase (PI3K)—AKT, and myocardin-related transcription factor (MRTF)^70,71^. The inhibition of MRTF^72^, and the activation of retinoic acid receptor (RAR)-α^73^ and RAR-γ^74^ have been shown to attenuate subretinal fibrosis by supressing TGF-β signalling^75^. Disruption of junctional proteins including ZO-1 increases RPE cell proliferation and cell dedifferentiation, in turn inducing EMT^76^. A similar process may be occurring in the early stages in JR5558 mice, and the resulting change in the RPE as well as damage of photoreceptor was confirmed in later stages. Further analysis of EMT inducers important in EMT, including Twist, Snail, and Slug^77^, at the early time points of 4 and 6 weeks will add mechanistic insights into the progression of subretinal fibrosis in JR5558 eyes.

While gliosis can be neuro- and vasculo-protective, it can also contribute to vision threatening scar formation^7^. During disease states, reactive Müller cells and astrocytes produce ECM in their involvement in retinal repair^19^. A study of posttranslational modification of GFAP polymers found they are accumulated and citrullinated within Müller cell processes and endfeet in both human wet-AMD and JR5558 mice retinal tissues^44^. Further, protrusions of Müller processes into the subretinal space has been reported to form fibrotic tissue that blocks the regeneration of outer segments but not anatomic reattachment^78,79^. We show GFAP^+^ Müller glia within the lesion sites are positive for vimentin and CRALBP, indicating their reactive state. However, gliosis was not noted in all subretinal lesions assessed by retinal section analysis. As the gliotic processes may be located outside of the sectioned area we also assessed gliosis in flatmount stained posterior cups. Interestingly, the same result was noted with not all subretinal fibrosis lesions associated with GFAP positive gliosis in the subretinal space. This could be due to ‘maturity’ of the subretinal lesion, having only recently been formed, or an indication that the lesion(s) may have resolved without forming a fibrotic scar. This was counter to what we expected but confirms GFAP to be an indirect marker of fibrosis^80^.

Pericytes, supportive peri-vascular cells that wrap the vascular endothelium, have been implicated in the progression of fibrosis by transdifferentiating into myofibroblasts^81^. Interestingly, the glutamate-aspartate transporter (GLAST), a major glutamate uptake carrier within Müller cells^82,83^, is also noted in type-A pericytes where it is utilised to distinguish these from non-scar-forming perivascular cells^84^. Type-A pericytes are recruited to mediate wound healing following tissue damage and consequently form a fibrotic scar for axons to regenerate following spinal cord injury^85^. Genetic ablation of type-A GLAST^+^ pericytes has shown to significantly diminish fibrotic scar formation in a model of spinal cord injury^86^. We noted specific GLAST expression within the fibrotic lesion site that align with pericytes where the distinct clear pattern of GLAST^+^ gliosis was not evident.

The subretinal space is an immune privileged site devoid of immune cells under homeostatic conditions. Retinal microglia and the complement system, as the first line of retinal defence, are activated to participate in the wound healing process. Prolonged chronic low-grade activation of retinal microglia^87^, complement activation and choroidal macrophage infiltration^88,89^, cause collateral damage to the surrounding cells of the subretinal space, stimulating glial proliferation and ultimately resulting in subretinal fibrosis. Analysis of drusen deposits in AMD show they contain lipids, proteins and complement products, indicating a primary role of an overactive immune response in AMD pathogenesis^90,91^. The pathogenesis of subretinal fibrosis, even if partially understood, consists of leukocyte exudation by the highly permeable new vessels, which in turn initiates local inflammation. Infiltrating macrophages can transdifferentiate into myofibroblasts through TGF-β and the complement component C3a^89^. We found increased microglial activation within the retina as well as infiltration of Iba-1 positive microglia/ macrophages in the subretinal space in JR5558 mice. Retinal microglia are yolk sac-derived and highly specialised immune cells that share characteristics of macrophages, and are able to migrate and accumulate in the subretinal space and adhere to the RPE in light induced retinal degeneration^92^. Additional analysis of these cells located in the subretinal space is required to determine whether they represent microglia that have migrated to this area or are monocyte-derived macrophages; a recent report suggests surface markers are enough to distinguish the two populations^93^. The presence of these cells in the subretinal prior to lesion development may also indicate that a similar macrophage to myofibroblast transition plays a central role in subretinal fibrosis development in the JR5558 line.

Laying of excessive collagen by myofibroblast can occur via transdifferentiation of any of the cell types mentioned. Further investigation is required to determine which subpopulation and the specific contribution is involved in ECM deposition in the subretinal space leading to subretinal fibrosis. A CRISPR/Cas9 approach similar to a recent publication assessing the role of macrophages in collagen deposition in scar formation within the heart^94^ can be utilised to visualise and determine the specific role of individual cell populations in subretinal fibrosis. Single-cell proteomics, RNA sequencing and systems biology approaches in investigating subretinal lesions will allow insight into the underlying mechanisms, including the signalling pathways activated and cells involved, behind the pathogenesis of subretinal fibrosis.

Very-low-density lipoprotein receptor (VLDLR) deficient mice (Vldlr-/-) that have a knockout of the gene encoding VLDLR is another mouse strain exhibiting consistent retinal angiogenesis and subretinal neovascularisation^95^. VLDLR has been shown have a nominal correlative association in family-based or case–control AMD datasets^96^, and the retinal neovascularisation dependent on LRP5 signaling^97^. VLDLR expression was found within the RPE, ganglion cell layer vessels, and the outer limiting membrane, with VLDLR knockout animals presenting neovessel growth originating from retinal vessels and progressing to the subretinal space, and leading to RPE disruption as well as photoreceptor degeneration^98^, as well as focal activation of Müller cells and upregulation of VEGF and GFAP^99^. Vimentin-positive fibroblasts were noted at 12 months in retinal lesion sites^98^ and Cx3cr1-positive microglia/macrophages were present in the outer retina and associated with subretinal neovascular angiomas of Vldlr-/- mice^100^. Importantly, fundus images reveal Vldlr-/- mice do not present subretinal lesions that are as bright as the JR5558 mice^97^ with retinal fibrosis not noted in significant amounts until 12 months of age^98^, thereby limiting the translational value of this model for studies on how to prevent subretinal scars.

The JR5558 mice model has some inherent limitations in investigating retinal fibrosis. The absence of the macula in mice means that the JR5558 model are not able to be used to investigate formation and progression of macular fibrosis. Analyses of a pure RPE monolayer was not undertaken in this study as it required pooling from many mice to yield enough protein to perform the necessary WB analyses to assess specific changes in the RPE, placing this outside the scope of our analysis. Another limitation is that the posterior cups, retinae, and choroids of 9-week C57BL/6J mice were used as controls to compare ECM and fibrotic markers in our WB analyses. The normal steady state retina does not undergo gliosis, extensive ECM remodelling, neovascularisation, and does not develop retinal fibrosis as is clearly shown in the retinal imaging we present which is the main outcome of the paper. Indeed, differences in many different components of the normal neural retina, including presynaptic photoreceptor ribbon synapses, GFAP^+^ retinal gliosis and CD11b^+^ microglia activation were noted in wild-type mice^101^. Taken together with technical limitations, including the limited number of lanes in WB gels run along with the requirement of enough biological replicates in each run to rigorously assess significance, meant that age-matched controls for all timepoints analysed (4-, 8-, 12-, and 20-week) could not be assessed in our study. Whilst both sexes of animals were utilised in this study, breeding resulted in higher number of males born, and hence each series of experiments included a higher proportion of males than females. Therefore, the influence of sex hormones on subretinal lesion formation and progression, and response to IVT treatment, could not be assessed. Finally, as a large range of variation in lesion formation was noted at 4 weeks in this transgenic line (Fig. 10 and Supplemental Fig. 5), care needs as taken to ensure proper distribution of animals with higher-than-normal lesion numbers at 4 weeks when assessing efficacy of treatment groups in ameliorating fibrotic lesions.

Taken together, our findings indicate that JR5558 mice develop spontaneous subretinal lesions with a fibrotic component that grows reliably and predictably between 4 and 8 weeks of age. Accordingly, the JR5558 mouse line may be a good model to further study the mechanisms of subretinal fibrosis, the contribution of specific cell populations in its pathogenesis and strategies for the prevention of subretinal fibrosis.

## Materials and methods

### Animals

This study was conducted in accordance with the Association for Research in Vision and Ophthalmology Statement for the Use of Animals in Ophthalmic and Visual Research and approved by the University of Sydney Animal Ethics Committee. All animals were housed in pathogen-free facilities on a 12 h light/dark cycle and were fed rodent chow (Diet T2920x, Envigo, IND, USA). JR5558 mice (also termed neoretinal vascularization [NRV]-2)^25^ were purchased from Jackson Laboratories (B6.Cg-*Crb1*^*rd8*^* Jak3*^*m1J*^/Boc: JAX stock# 005,558). C57BL/6J mice served as controls, with animals of both sexes included in all studies, unless stated otherwise. The animal study reporting in the manuscript follows the Animal Research: Reporting of In Vivo Experiments (ARRIVE) guidelines.

### In Vivo* imaging*

Colour fundus photography (CFP), optical coherence tomography (OCT) and fundus fluorescein angiography (FFA) were performed with the MICRON IV Retinal Imaging Microscope (Phoenix Technology Group, Pleasanton, CA). The pupils of mice anaesthetized with ketamine (48 mg/kg) and medetomidine (0.6 mg/kg) were dilated with a topical drop of 1% Tropicamide and 2.5% phenylephrine hydrochloride. Then real-time fundus image-guided OCT was performed on both eyes in accordance with the manufacturer’s image acquisition software, with the patterns of retinal vasculature used as a key marker to image the same area in consecutive imaging experiments, with the optic nerve located at the centre of images. FFA was conducted in both eyes from 30 to 5 min after fluorescein injection, as previously described^25^.

### Hematoxylin and Eosin and Picro-Sirius Red staining of paraffin retinal sections

Eyes were nucleated from JR5558 mice aged 12–16 weeks and fixed in 4% paraformaldehyde (PFA) for 1 h at room temperature (RT), embedded in paraffin and sectioned. Retinal sections of 10 μm thickness underwent histological staining with hematoxylin and eosin (H&E) and Picro-Sirius Red (PSR) staining protocol^102^ to study changes in retinal morphology and expression of connective tissue (collagen), respectively. Image were captured with an Axioplan 2 (Zeiss, Oberkochen, Germany) light microscope.

### Immunostaining of frozen sections and retinal flatmounts

Enucleated eyes from 12 to 16 weeks JR5558 and C57BL/6J mice (unless otherwise stated) were fixed in 4% PFA for 1 h at room temperature (RT), placed in 30% sucrose at 4 °C overnight, embedded in O.C.T (Tissue-Tek O.C.T; Sakura#4583) and sectioned at a thickness of 10 μm with a cryostat. Frozen sections were blocked with (10% Normal Donkey Serum for 1 h at room temperature), washed (3 × 5 min) with 0.05% Tween-20 in PBS, and incubated in staining solution containing 1% FBS (Sigma #F9423) and 1% Triton-X100 (Merck #30,632.4 N), followed by incubation with antibodies (listed in Table 2), at 4 °C overnight. Frozen sections were then washed, incubated with Alexa Fluor 488 or 594 conjugated secondary antibody solution (1:1000; Invitrogen) for 3 h at RT, counterstained with Hoechst and mounted on polysine coated microscope slides (Menzel-Gläser, Braunschweig, Germany) with Vectashield Antifade Mounting Medium (Vector Laboratories Inc., Burlingame, CA).

**Table 2:** Antibodies used for immunohistochemistry (IHC), immunocytochemistry (ICC) and western blots (WB).

Antibody	Source and Catalogue No	Dilution (IHC or ICC)	Dilution (WB)
CD31	BD, #550,274	1:50	N/A
Collagen I	Abcam, #ab34710	1:100	1:2000
Collagen IV	BioRad #2150–1470	1:200	N/A
Cone Arrestin	Millipore, #AB15282	1:500	1:2000
CRALBP	Abcam, #15,051	1:100	N/A
CTGF	Abcam, #125,943	1:200	1:1000
Cytokeratin 19	Abcam, #52,625	1:200	N/A
Fibronectin	Chemicon, #AB2033	1:100	1:1000
GFAP	Dako#Z0334	1:250	N/A
	Cell Signalling Technology, #3670	N/A	1:1000
GLAST	Chemicon (Millipore)	1:200	–
HES1	Cell Signalling Technology, #11,988	N/A	1:1000
HES5	Millipore, #AB5708	N/A	1:500
Iba-1	Wako, #019-19,741	1:500	N/A
IB4-594	Invitrogen, #I21413	1:50	N/A
Integrin α5	Cell Signalling Technologies, #98,204	N/A	1:1000
MMP2	NovusBio, #AF1488-SP	N/A	1:500
MMP9	NovusBio, #AF909	N/A	1:200
PEN2	Cell Signalling Technologies, #5898	N/A	1:1000
PNA-488/594	Invitrogen, # L-21409/ 32,459	1:100	N/A
Presenilin 1	Cell Signalling Technologies, #4053	N/A	1:1000
Rhodamine Phalloidin	Life Technologies, #R415	1:40	N/A
Rhodopsin	Chemicon, #MAB5356	1:200	N/A
RPE65	NovusBio, #NB100-355	1:200	N/A
Smad3	Cell Signalling Technologies, #8685	N/A	1:1000
Stat3	Cell Signalling Technologies, #4904	N/A	1:2000
TGFβ-pan	Cell Signalling Technologies, #3711	N/A	1:1000
TGFβ-RI	Abcam, #31,013	N/A	1:1000
Vimentin	Abcam, #92,547	1:250	1:1000
α-SMA	Cell Signalling Technologies, #48,938	1:200	1:1000

For retinal/RPE flat mounts, dissected eye cups from 12 to 16 weeks JR5558 mice (unless otherwise stated) were fixed in 4% paraformaldehyde for one hour at RT and then placed in PBS at 4 °C. The neural retina and RPE attached to the choroid/scleral complex were separated. Neural retinas were immersed in 30% sucrose overnight and repeatedly shock-frozen and thawed to improve antibody penetration. Following 3 washes, retinas were then incubated with antibodies against GFAP, CD31, and Iba-1 (see Table 2 for details) in staining solution containing 1% FBS and 1% TritonX-100 for 3–5 days. Flat mounts were then washed and incubated in Alexa Fluor 488 or 594 conjugated secondary antibody solution (1:1000; Invitrogen) for 3 h at RT, counterstained with Hoechst, and imaged with an upright LSM700 confocal microscope (Zeiss).

Retinal sections and flatmounts were imaged with an upright LSM700 confocal microscope (Zeiss), and images processed with ImageJ or Imaris (Bitplane, Zurich, Switzerland) software.

### Western blots

The neural retina and RPE with the choroid/scleral complex were separated, snap frozen in liquid nitrogen and stored at − 80 °C until all samples had been collected. RPE-choroid-scleral complexes from two eyes were pooled to prepare one sample for analysis of changes in RPE proteins, whereas neural retinas were individually assessed. Samples were processed as previously described^103^. To summarise, following lysis with RIPA buffer (Sigma #R0278) and Protease/Phosphatase Inhibitor Cocktail (Cell Signalling #5872), protein concentrations were determined by QuantiProTM BCA Assay Kit (Sigma#QPBCA-1KT) protein assay and equal amounts of protein were subjected to SDS–polyacrylamide gel electrophoresis with NuPAGE Tris‐Bis gel (Life Technologies, Mulgrave, Australia). The gels were then transferred to a polyvinylidene difluoride (PVDF) membrane, the gel probed with primary Abs (Table 2) overnight at 4 °C, then incubated with secondary Abs conjugated with horseradish peroxidise for 2 h at room temperature. Protein bands visualised using the G:Box BioImaging system were quantified using the GeneTools image scanning and analysis package and expression normalized to β-tubulin (rabbit polyclonal, 1:2000; Cell Signalling #2148).

### Lesion area quantification

CFP images were quantified in a semi-automated manner using an open-source platform FIJI/ ImageJ (http://imagej.nih.gov/ij/, National Institutes of Health, Bethesda, MD)^104^. Images were converted to 8-bit and underwent background subtraction with a rolling ball radius of 200 μm (Supplemental Fig. 1A,B). Lesions that were totally or partially within a circle with a radius of 283 μm from the optic nerve, signifying the peripapillary region, and equating to approximately half of the fundus area imaged, were selected with the line/freehand line tool (Supplemental Fig. 1C). The optic nerve being at the centre of CFP images ensured that the lesions captured in the peripapillary region assessed were enface, and the accuracy of lesion area quantified. The area outside the selection was cleared (Supplemental Fig. 1D) and the image adjusted with thresholding. The resulting binerised image was checked and any necessary closure or addition/ removal of pixels made (Supplemental Fig. 1E). The particles within the image were then analysed by ImageJ, and the number and lesion area recorded. The resulting lesion regions was then confirmed against the original fundus image (Supplemental Fig. 1F). In experiments where multiple fundus images were captured from the same animal over time, the region number assigned to each lesion area was also recorded to ensure the same lesion was tracked over time.

### Intravitreal Aflibercept treatment

Following in vivo imaging, a single intravitreal injection with 2 mg of Aflibercept (Eylea) was performed anaesthetised JR5558 mice. JR5558 mice receiving sham intravitreal injection of sterile PBS served as controls. In vivo imaging was again performed 2 weeks post treatment and CFP, OCT FFA images analysed as above.

### Statistical analysis

Results are expressed as mean ± SEM. Graphpad (Prism, version 8) and SPSS 17.0 (Windows) software were used for the statistical analysis. Data were analysed using unpaired student t-test and one-way ANOVA with a post-hoc (Bonferroni’s or Dunnett’s) correction to analyse significant differences among groups. A *p*-value < 0.05 was regarded as statistically significant.

### Supplementary Information

Supplementary Figure 1.

Supplementary Figure 2.

Supplementary Figure 3.

Supplementary Figure 4.

Supplementary Figure 5.

Supplementary Video 1.

## Data Availability

The data that support the findings of this study are available in the methods and body of text and can be made available by the corresponding authors upon request.
