# Supplementary figures and images for: JR5558 mice are a reliable model to investigate subretinal fibrosis
Source: Sci Rep. 2024 Aug 13;14:18752. doi: 10.1038/s41598-024-66068-z (PMC11322289; doi:10.1038/s41598-024-66068-z)

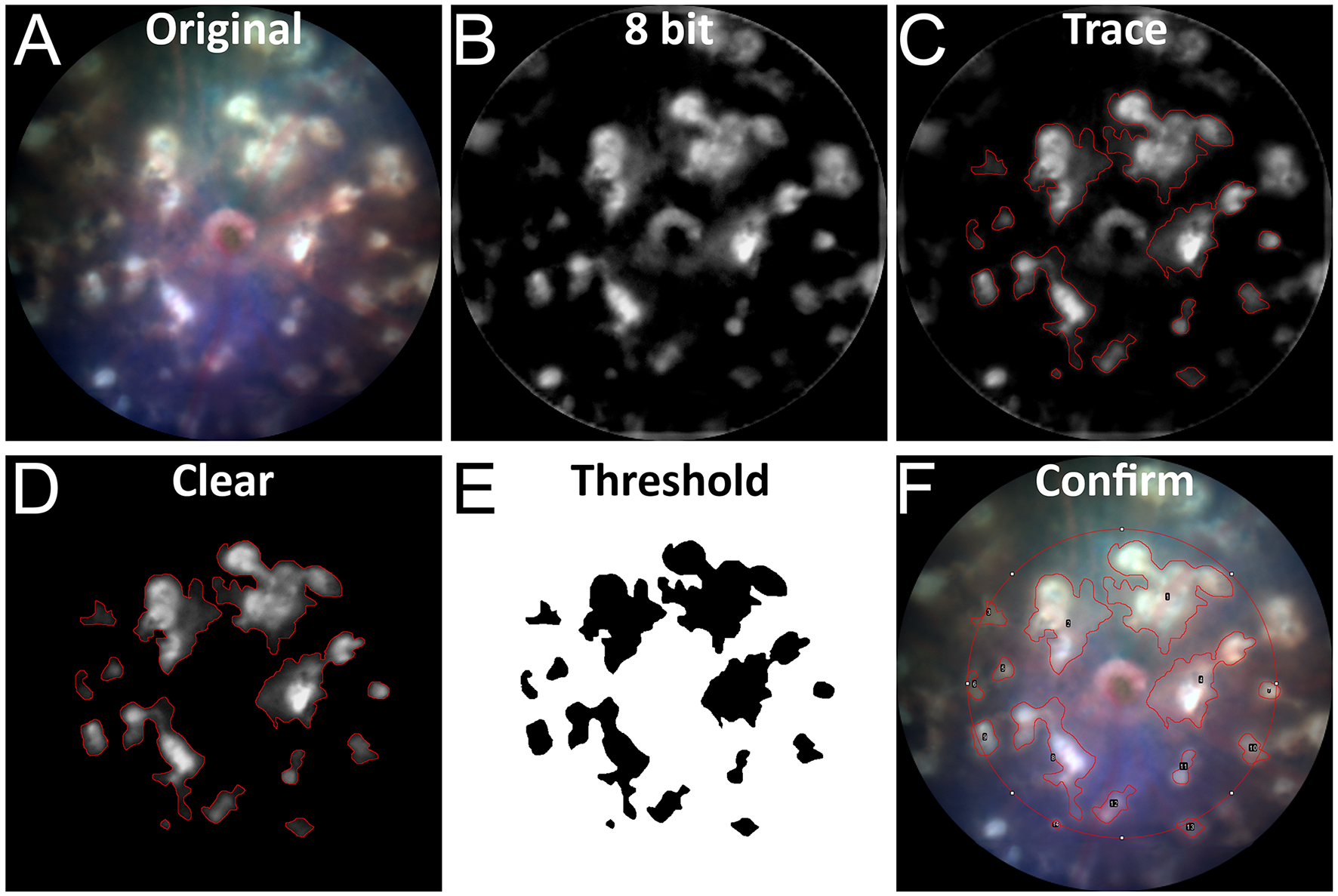

Supplement: Supplementary file 1 — Supplementary Figure 1. [file 41598_2024_66068_MOESM1_ESM.tif]

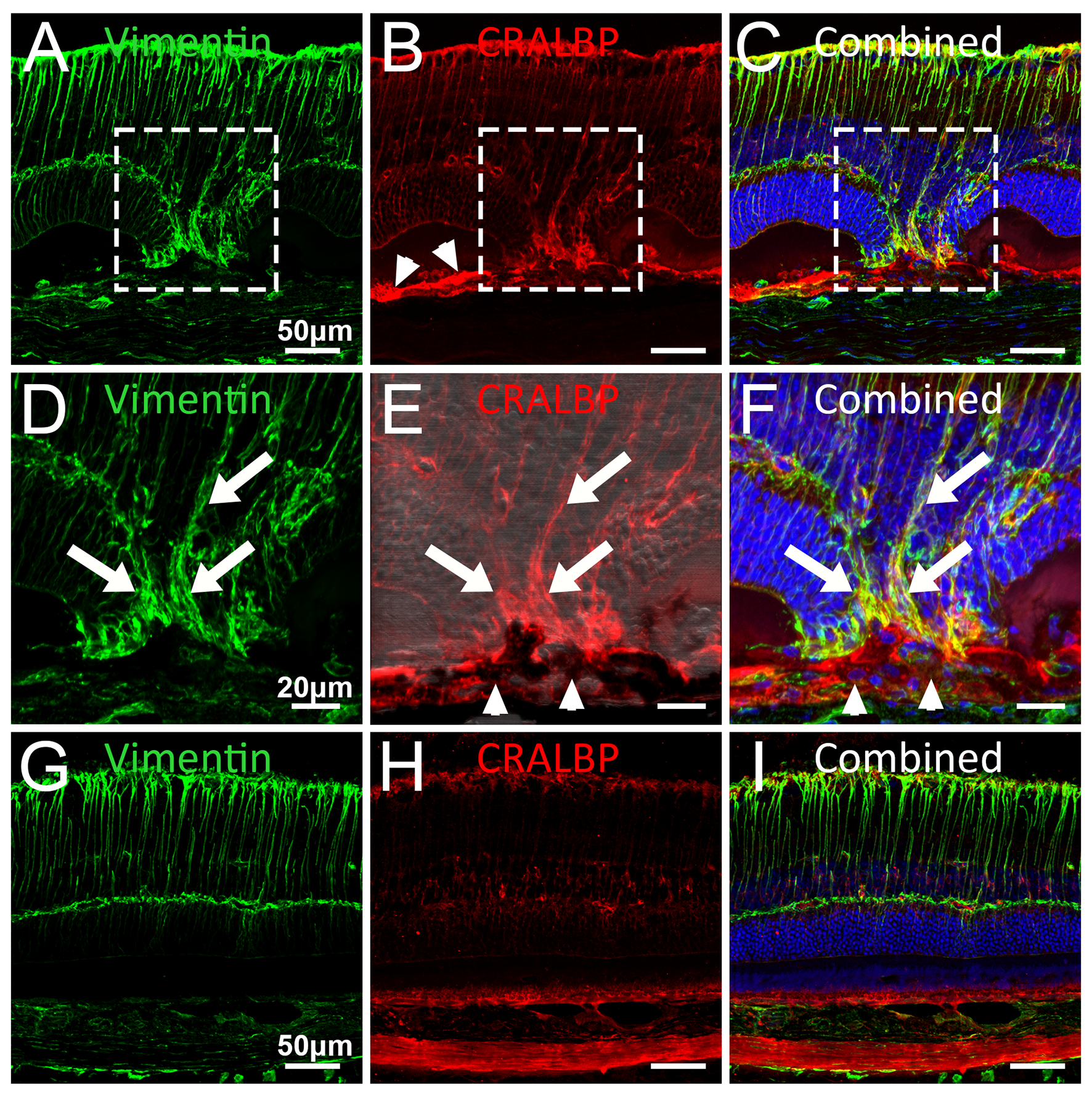

Supplement: Supplementary file 2 — Supplementary Figure 2. [file 41598_2024_66068_MOESM2_ESM.tif]

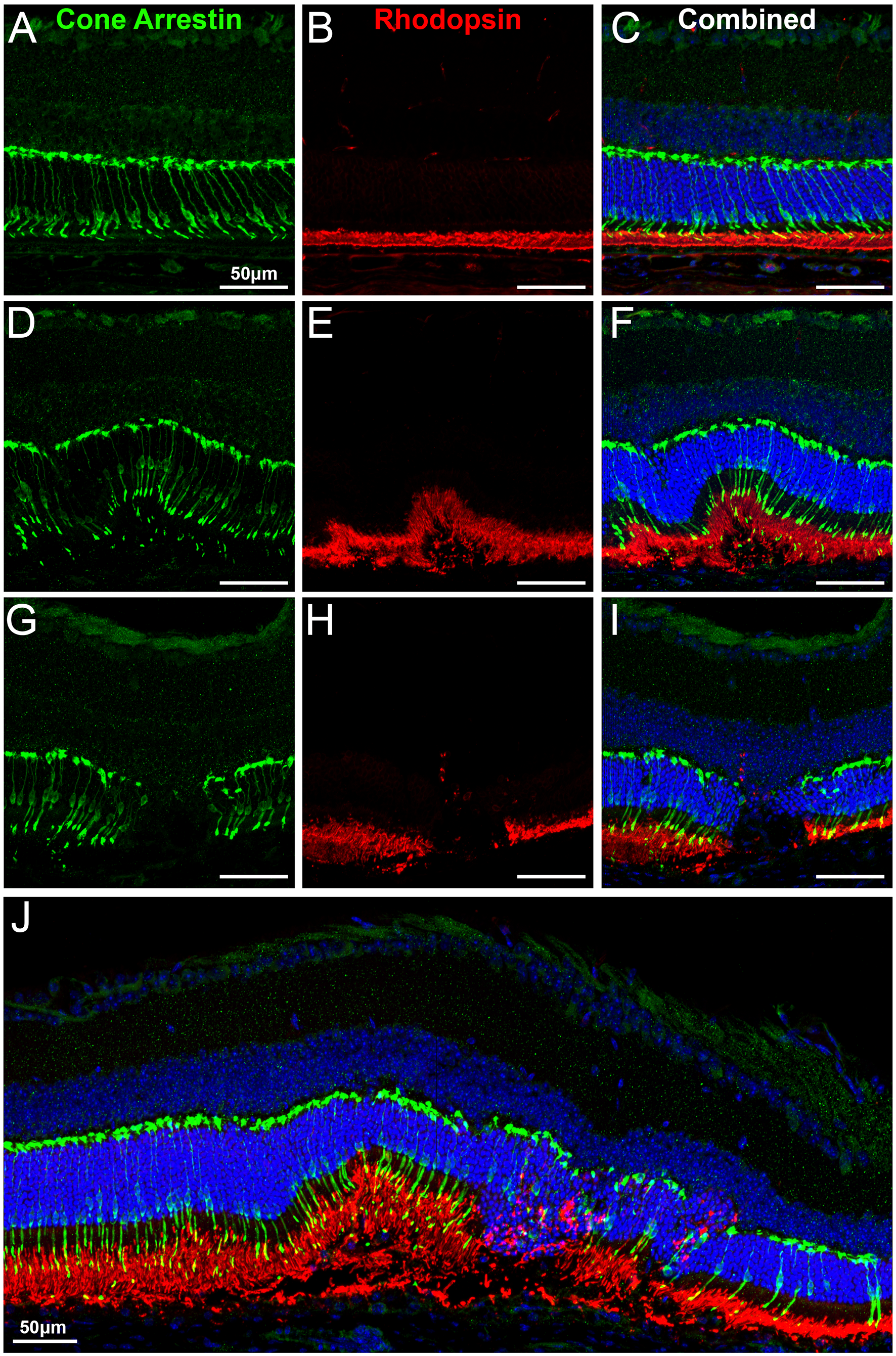

Supplement: Supplementary file 3 — Supplementary Figure 3. [file 41598_2024_66068_MOESM3_ESM.tif]

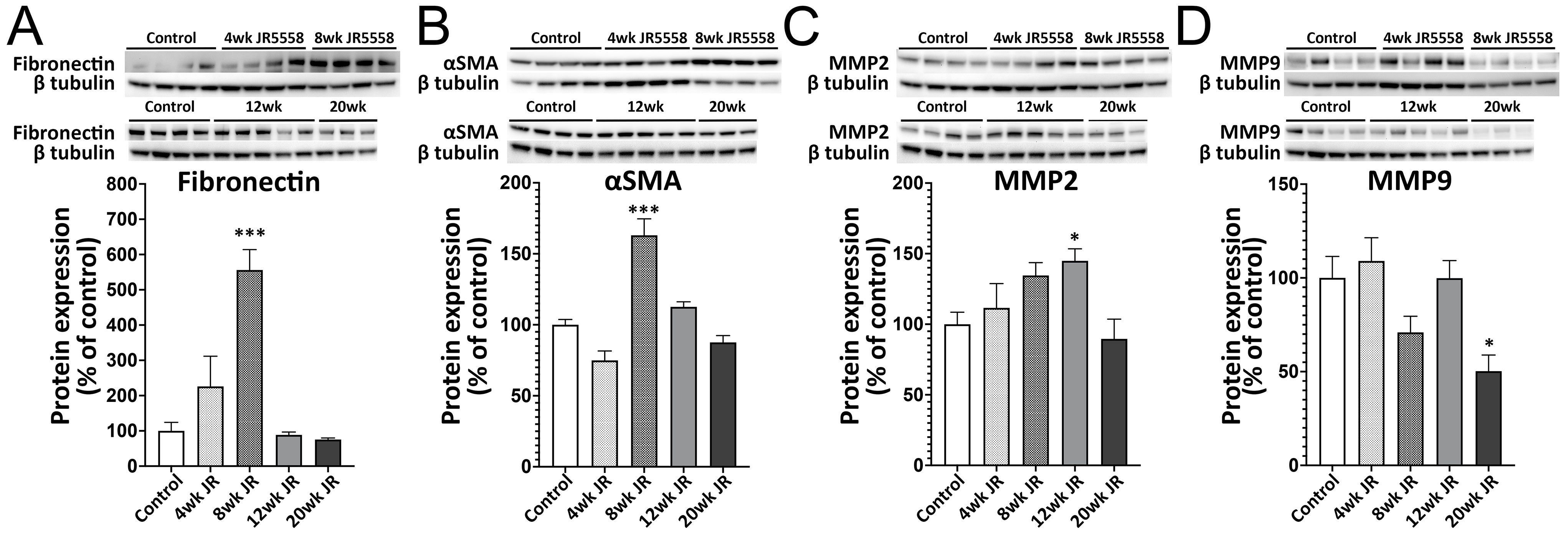

Supplement: Supplementary file 4 — Supplementary Figure 4. [file 41598_2024_66068_MOESM4_ESM.tif]

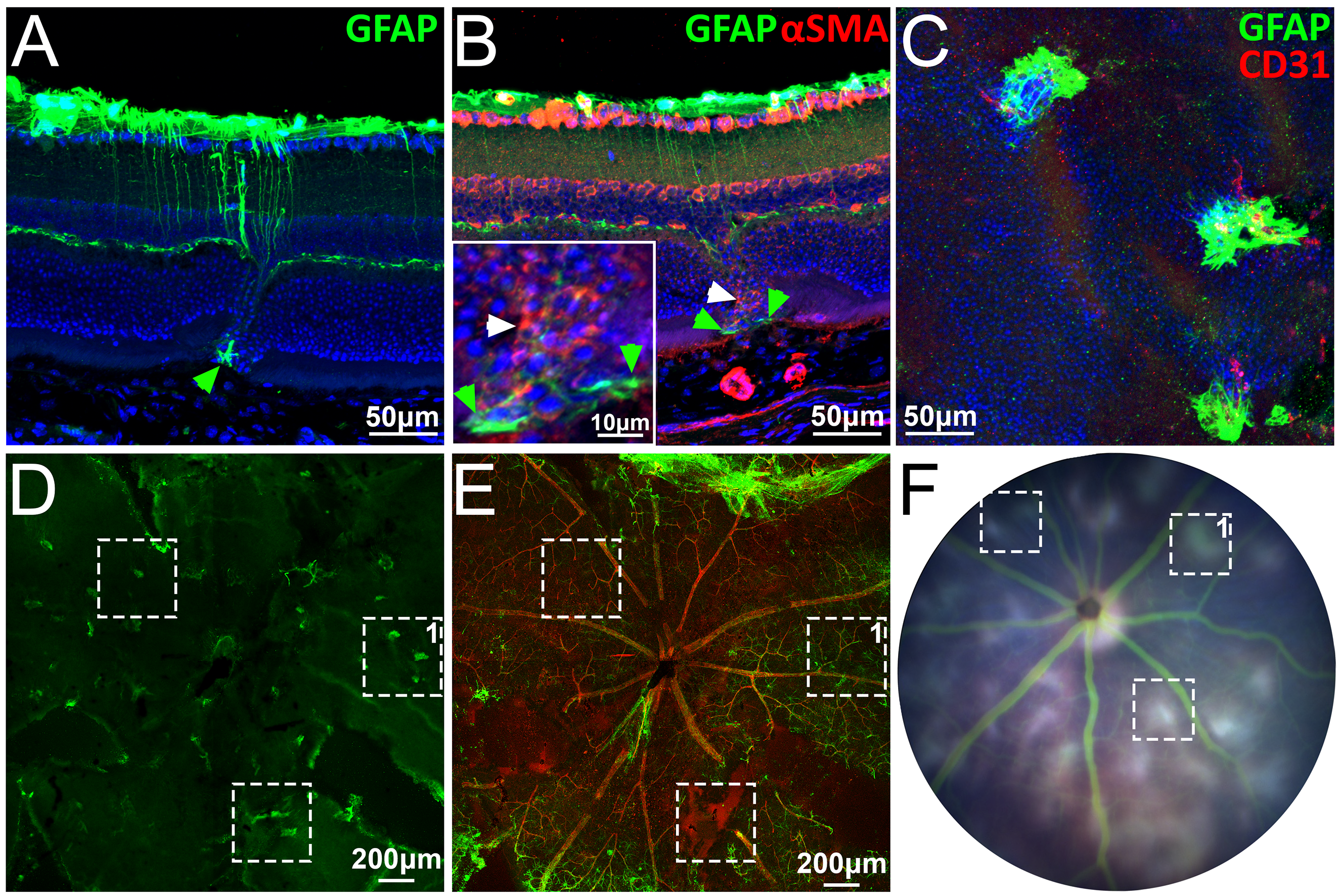

Supplement: Supplementary file 5 — Supplementary Figure 5. [file 41598_2024_66068_MOESM5_ESM.tif]
